# The Effects of High-Intensity Multimodal Training in Apparently Healthy Populations: A Systematic Review

**DOI:** 10.1186/s40798-022-00434-x

**Published:** 2022-03-29

**Authors:** Tijana Sharp, Clementine Grandou, Aaron J. Coutts, Lee Wallace

**Affiliations:** Sport and Exercise Discipline Group, University of Technology, Sydney Human Performance Research Centre, Moore Park, Sydney, Australia

**Keywords:** High-intensity, Multimodal training, Aerobic fitness, Muscular fitness, Subjective responses

## Abstract

**Background:**

High-intensity multimodal training (HIMT) is emerging as a popular training method that combines aerobic and resistance training throughout a single exercise session. The current literature is limited by a lack of terminology that broadly encompasses all styles of combined aerobic and resistance training. The magnitude of chronic or long-term (i.e.  ≥ 4 weeks) effects of HIMT participation on aerobic and muscular fitness also remains unclear. Additionally, one of many complex reasons for the growing popularity of HIMT may be attributed to the affective response to exercise, namely levels of enjoyment. However, this concept is not yet well understood across all styles of HIMT. A comprehensive systematic review is required to synthesise the available literature and attempt to provide an operational definition of HIMT to capture the breadth of combined training styles that exist.

**Objective:**

The objective of this systematic review was to determine the chronic effects of HIMT participation on aerobic and muscular fitness and to compare HIMT to established concurrent training methods. Enjoyability and other adherence-related subjective responses were also examined in HIMT participants. This review critically assessed the level of evidence and feasibility of current HIMT guidelines.

**Methods:**

A systematic literature search was conducted on PubMed, Web of Science and SPORTDiscus to identify studies up until March 2021.

**Results:**

A total of 20 studies were included for review. Studies generally reported moderate to large effects on aerobic fitness and subjective responses in favour of HIMT interventions. Mixed outcomes were demonstrated in muscular fitness. These results should be treated with caution due to high risk of bias among included studies.

**Conclusions:**

Few studies have assessed the chronic effects of HIMT participation on aerobic, and musculoskeletal adaptations and subjective responses, in particular exercise enjoyment. Research conclusions are limited by heterogeneity of experimental protocols and outcome measures. Furthermore, the inability of the literature to make adequate comparisons between various styles of HIMT and other concurrent training protocols limits understandings of the efficacy of HIMT.

*Registration* This systematic review was registered on the Open Science Framework (10.17605/OSF.IO/2RE4B; 26 March 2021).

**Supplementary Information:**

The online version contains supplementary material available at 10.1186/s40798-022-00434-x.

## Key points

- Previous studies have demonstrated positive effects of chronic high-intensity multimodal training (HIMT) participation on aerobic fitness and subjective responses in healthy adults.
- Mixed outcomes have been demonstrated in muscular fitness (e.g., strength, endurance, power).
- Disparate styles of HIMT and unstandardised reporting of interventions inhibit the ability to make clear comparisons with other concurrent training methods, limiting the understanding of the efficacy of HIMT.
- The subjective response to HIMT is not clearly understood. However, previous findings for high-intensity interval training (HIIT) show that HIMT may be enjoyable, which has implications for promoting exercise adherence.

## Introduction

**Fig. 1:**
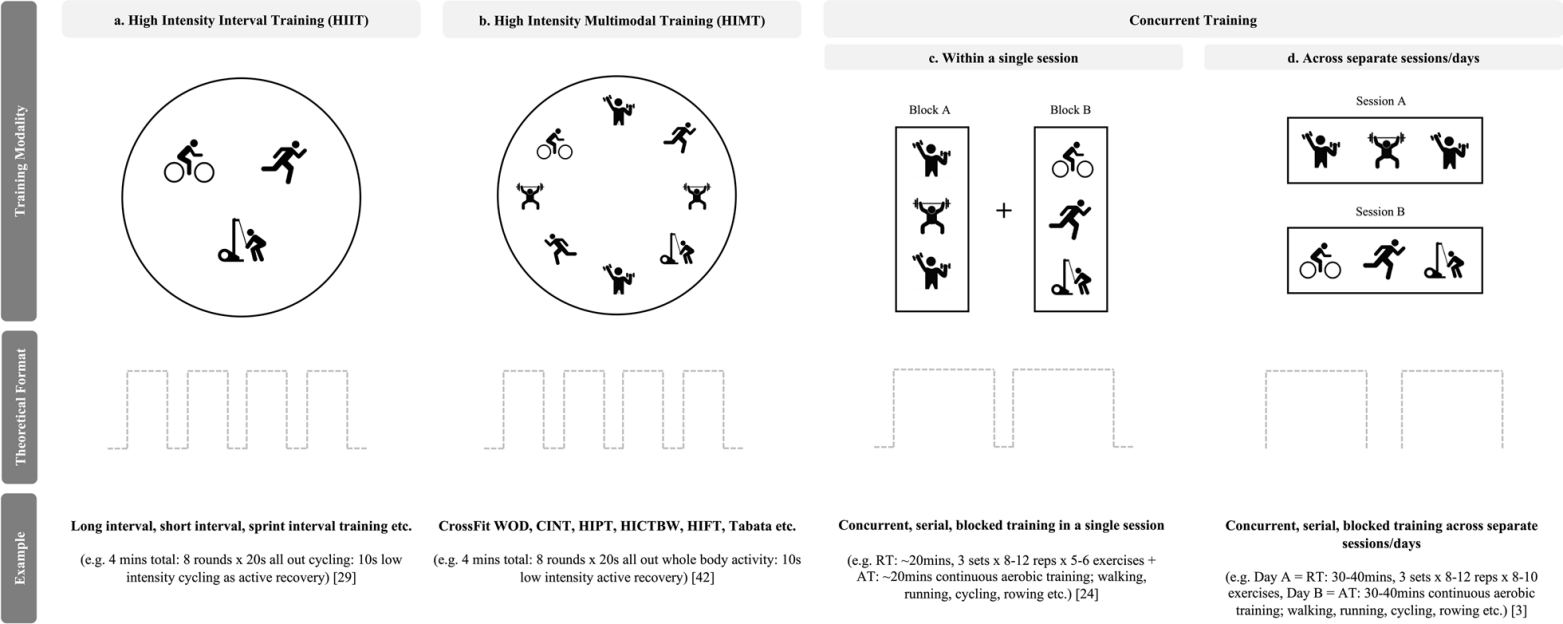
Description of relevant training modalities in the literature. *HIIT* high-intensity interval training, *HIMT* high-intensity multimodal training, *WOD* workout of the day, *CINT* circuit-type neuromuscular exercise training, *HIPT* high-intensity power training, *HICTBW* high-intensity circuit training with bodyweight, *HIFT* high-intensity functional training, *RT* resistance training, *AT* aerobic training

Regular participation in physical activity is known to positively influence various outcomes including physical and psychological health, well-being and quality of life [1, 2]. Physical activity guidelines recommend that healthy adults participate in both aerobic (i.e. ≥ 30 min of moderate intensity on 5 days/ week or ≥ 20 min of vigorous intensity on 2 days/ week) and resistance-based exercise (i.e. ≥ 2 days/ week) to reduce the risk of morbidity and mortality [3]. However, adherence to physical activity guidelines remains low, with lack of time (to accumulate both aerobic and resistance training) and poor exercise enjoyment or intrinsic motivation among the most commonly reported barriers to exercise participation [4–8]. Combining aerobic and resistance training modalities into a single time-efficient exercise session may help individuals fulfil the current physical activity recommendations and has recently gained interest [9, 10]. For example, high-intensity interval training (HIIT), bodyweight, functional fitness and group training are among the top 20 worldwide fitness trends for 2021 [10]. There have been recent attempts to label and define this emerging training trend using terms such as high-intensity functional training (HIFT), CrossFit®, bodyweight HIIT, resistance HIIT and circuit high-intensity interval training [9, 11] (Fig. 1b). However, operational terms such as functional, bodyweight and resistance may not consistently capture all combinations of aerobic and resistance modalities or may be confused with goals associated with motor learning and performance [12, 13]. Namely, the term functional can be used interchangeably as a movement or modality description. Additionally, previous definitions are limited by large variation in exercise prescription (i.e. modality, intensity, work-to-rest ratio). Therefore, for the purposes of this review the authors introduce the term high-intensity multimodal training (HIMT) as a broader defining term that encompasses all relevant styles of combined aerobic, resistance and/ or bodyweight training (i.e. HIFT, bodyweight HIIT, CrossFit®) performed at a high or vigorous intensity (Fig. 1b). High or vigorous exercise intensity is defined by the American College of Sports Medicine (ACSM) [3, 14] as activity that sustains:

-  > 77% heart rate maximum (HRmax) OR 80–90% HRmax during work periods and 40–50% HRmax during active or passive rest periods;
- Rating of perceived exertion (RPE) > 14 out of 20;
-  > 70% 1 repetition maximum (1RM);
- Or an inability to speak more than a few words.

HIMT may contain similar components of HIIT which typically involves a single aerobic exercise mode (i.e. cycling or running) (Fig. 1a, b). Both may be characterised by repeated bouts of high- or vigorous-intensity activity interspersed with periods of active or passive rest [15]. The current popularity of HIIT training can be largely attributed to its ability to elicit significant training adaptations in a time-efficient manner [11, 16]. Similarly, HIMT may be an attractive exercise method for accumulating aerobic and resistance training into one session. Previous studies have attempted to investigate the chronic or long-term (i.e. ≥ 4 weeks) health and fitness outcomes of HIMT [17–20]. These studies suggest that the combination of aerobic and resistance training stimulus of HIMT elicits time-efficient aerobic and muscular fitness adaptations [21, 22]. However, due to the limited ability to prescribe, control and monitor high or vigorous levels of intensity and difficulties in standardising external work, it remains unclear whether HIMT is as effective as other concurrent aerobic and resistance training interventions [9], for example, concurrent training where aerobic and resistance exercise are distributed into separate training blocks within a single session (Fig. 1c) or on different days (Fig. 1d).

Positive subjective responses including increased intrinsic motivation and exercise enjoyment have been shown to promote greater exercise adherence and therefore may play a role in the improved health and fitness outcomes associated with HIMT [5–7]. The reasons for exercise initiation and adherence are complex and can be attributed to many interrelating environmental, social, cognitive, physiological and personal factors [23]. Among them, the affective response to exercise, in particular the experience of enjoyment, is suggested to impact exercise adherence by supporting intrinsic motivation [6, 7]. Previous investigations that have examined this association may offer possible explanations for the emerging popularity of HIMT [24–26]. The notion that HIMT might provide an enjoyable mode of training is primarily based on similarities with HIIT workouts, which have been shown to be more ‘enjoyable’ than steady-state modalities in healthy populations [27, 28]. However, high-intensity exercise has also shown to be painful and unpleasurable for individuals with poorer conditioning [29, 30]. To date, few studies have examined the effects of the various styles of HIMT on subjective responses such as enjoyment. A greater understanding of this relationship may provide insight into exercise behaviour and the impact of HIMT on exercise adherence. Therefore, the purpose of this review is to examine the effects of chronic HIMT participation on aerobic and muscular fitness and to compare HIMT to established concurrent aerobic and resistance training methods. Exercise enjoyment and other adherence-related subjective responses will also be examined in HIMT participants. This review will provide an operational definition of HIMT to effectively capture a broader range of combined aerobic and resistance training styles that currently are described in the literature. Finally, this review will assess the level of evidence of current HIMT training guidelines.

## Methods

This review was conducted according to PRISMA (Preferred Reporting Items for Systematic Reviews and Meta-Analysis) guidelines [31]. A systematic review protocol including the review question, search strategy, exclusion criteria and risk of bias assessment was prospectively registered with the Open Science Framework (10.17605/OSF.IO/2RE4B; 26 March 2021).

### Eligibility Criteria

Eligibility criteria were drafted and refined by three authors (TS, CG, LW) using exploratory searches. Included studies met the following criteria [population, intervention, comparator, outcome, study design (PICOS)] and report characteristics.

P: This systematic review included studies investigating the healthy adult population without contraindication to exercise only (over mean age 18 years—2 standard deviations and under mean age 65 + 2 standard deviations). Participants younger than 18 or older than 65 may show different adaptations to HIMT compared to adults and should be studied separately. If studies included both adults and the elderly or youth, they were included only if data reported for adults are reported separately. Studies examining participants with metabolic or chronic disease, musculoskeletal injuries or psychological disorders were excluded.

I: All longitudinal interventions (≥ 4-week duration) that primarily emphasise whole-body movements and combine aerobic and muscular training (resistance or bodyweight) into a single session. This may have included but was not limited to high-intensity functional training (HIFT), high-intensity circuit training (HIICT), multimodal training (MM-HIIT), high-intensity resistance training (HIRT) and CrossFit®. Accepted interventions elicited both cardiovascular and musculoskeletal training stimuli. Any protocols that did not specify a high, vigorous, all out or maximal intensity were excluded. Interventions that specified a high, vigorous, all out or maximal intensity but did not meet the ACSM guidelines for high-intensity activity were included to ensure literature saturation. Interventions that did not use HIMT as the sole exercise modality or used block or concurrent training where training modes are separated within sessions or on separate days were excluded.

C: Only studies with a comparator group were included in this review. Eligible comparators included at least one of the following:

- Passive control (no engagement in physical activity);
- Habitual activity control (continued regular physical activity habits);
- Structured activity control (structured activity control groups were included where aerobic and resistance training was completed in a concurrent format distributed into separate training blocks, either within (1) a single session or (2) on different days. No restriction was placed on exercise intensity).

O: The outcomes of this systematic review were reported health and fitness measures regarding:

- Aerobic capacity (e.g. oxygen uptake, heart rate variables);
- Muscular fitness (e.g. strength [1RM, grip strength], endurance [maximal repetition tests] and power [Wingate, peak power, counter movement jump]);
- Exercise enjoyment and other adherence-related subjective responses (e.g. HIIT self-efficacy, intrinsic regulation, identified regulation).

S: This search was limited to randomised parallel groups trials in order to ensure greater quality of included studies and meet the objectives of this review (comparison of HIMT with other concurrent training modalities). The search did not restrict publication status or language to ensure saturation of the literature. Potentially relevant studies not written in English were translated for assessment using Google Translate.

### Search

A search strategy was developed by three authors (TS, CG, LW). A literature search was conducted by a single author (TS) in PubMed, Web of Science Core Collection and SPORTDiscus. Sources were searched with no start date and an end date of March 2021. The Cochrane Central Register of Controlled Trials (CENTRAL) and sources of grey literature were also searched to ensure literature saturation. See Additional file 1: Table S1 for detailed search strategy and Boolean search string. No medical subject headings (MeSH) were used in this search strategy due to the non-clinical nature of the review. In addition to the database searches, the reference lists of relevant studies, reviews and books were screened for potential oversights. Relevant experts in the field were also consulted and their personal profiles searched to ensure saturation of the literature.

### Study Selection

Literature search results were exported into reference management software (Endnote X9), and all duplicate articles were removed. Articles were then imported into Covidence (Covidence Systematic Review Software, Veritas Health Innovation 2013) to assess eligibility. Two authors (TS, CG) independently screened the articles by title and abstract. All potentially eligible references proceeded to full-text screening. Conflicts were resolved by a third author (LW). Two authors (TS, CG) independently screened the full texts of all included records against the eligibility criteria. Conflicts were resolved by a third author (LW).

### Data Extraction

Two authors (TS, CG) independently extracted data from eligible studies. Data were imported into an Excel spreadsheet designed for this review (Additional file 2: Table S2). Extracted information included publication details (author, year), participant characteristics (sex, physical activity level/ training history), study methods (design), HIMT intervention (duration, mode, frequency, volume, intensity), comparator and sources of funding. Pre- and post-intervention measures (mean ± SD) and effect size were extracted for primary (aerobic and muscular fitness) and secondary (enjoyability) outcome measures. If pre- and post-intervention data were provided only in figures or not provided within the paper, the authors were contacted via email for further information. Where authors were uncontactable or did not respond [32–36], the online tool WebPlotDigitizer was used to manually extract data from the reported figures.

### Data Analysis

Effect sizes (Hedges’ g) and their 95% confidence intervals were calculated for all outcome measures of studies included in the review. The between-group effect sizes were calculated for HIMT intervention groups versus comparator group (i.e. passive or habitual activity control or structured activity [concurrent training]). To obtain the effect size change, scores (i.e. mean post–mean pre) were calculated for all groups and divided by the pooled standard deviation. To retrieve the pooled standard deviation, the change from baseline SD using a correlation coefficient of 0.8 was calculated using the formula recommended by the Cochrane guidelines [37]:

$${\text{SD}}_{{{\text{E}}\,{\text{change}}}} = \sqrt {{\text{SD}}_{{{\text{E}}\,{\text{baseline}}}}^{{2}} + {\text{SD}}_{{{\text{E}}\,{\text{final}}}}^{{2}} - \left( {2 \times {\text{Corr}} \times {\text{SD}}_{{{\text{E}}\,{\text{baseline}}}} \times {\text{SD}}_{{{\text{E}}\,{\text{final}}}} } \right)}$$

where SD_Ebaseline_ equals the standard deviation at the pre-test for the HIMT or comparator intervention, SD_Efinal_ equals the standard deviation at the post-test for the experimental or control intervention and Corr equals the correlation coefficient.

### Risk of Bias Assessment

The Cochrane ROB 2 tool for assessing the risk of bias for randomised trials was used to assess the possible risk of bias for each study (Table 8.5a in the Cochrane Handbook for Systematic Reviews of Interventions) [38]. This tool addresses sequence generation, deviation from intended interventions, incomplete outcome data, outcome measurement and selective reporting. For each domain, the methodology of each study was considered and a judgement of the possible risk of bias was made. Where there was insufficient information reported in the study, the risk of bias was judged as ‘unclear’ and the study authors were contacted for further information. Two reviewers (TS, CG) independently made these judgements per the Cochrane collaboration tool criteria. Conflicts were resolved by discussion. A third reviewer with experience in risk of bias assessments (FMI) reviewed all risk of bias evaluations. Unresolved conflicts were resolved by discussion.

## Results

### Study Selection

**Fig. 2:**
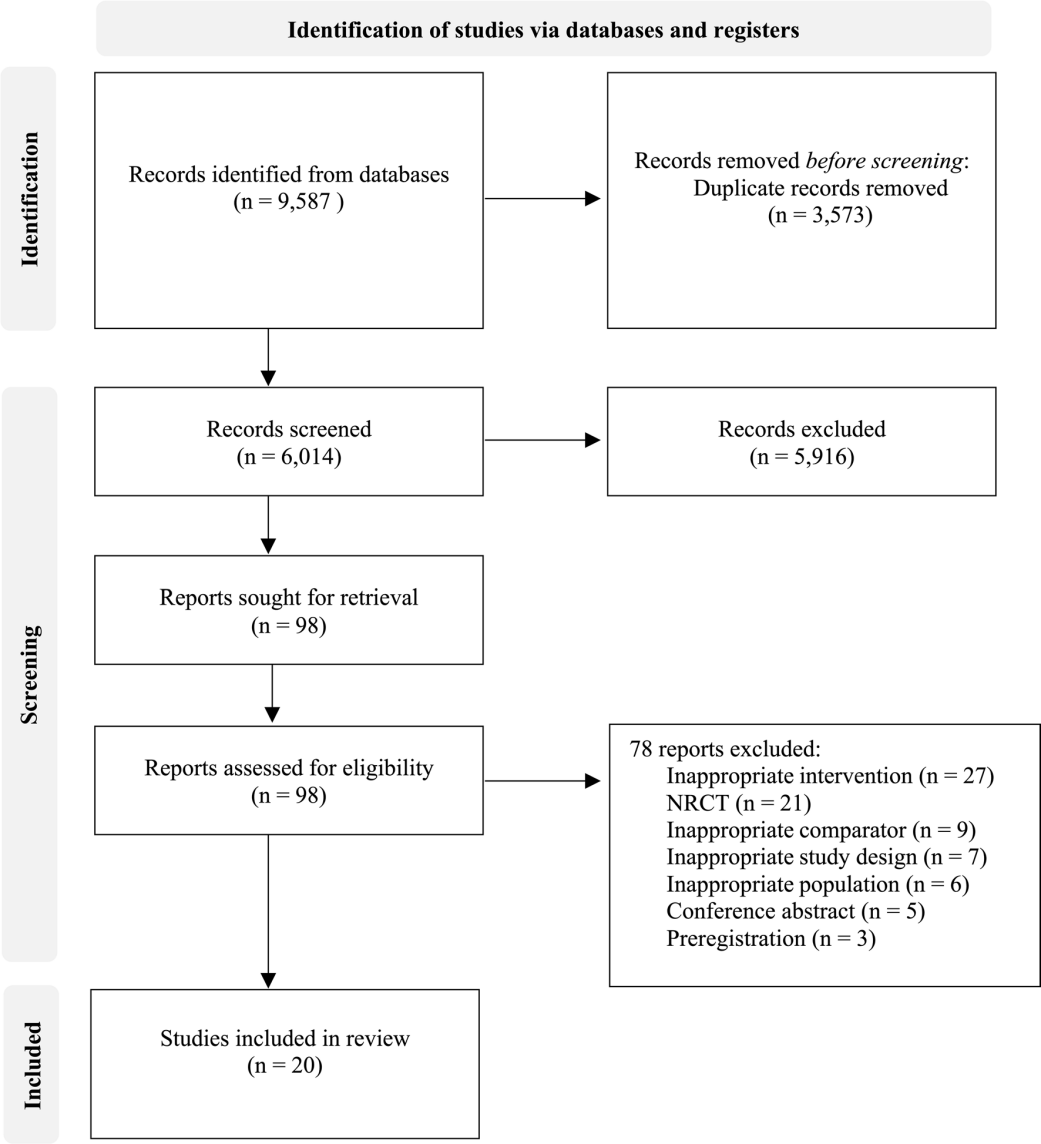
PRISMA flow diagram of systematic search and included studies *NRCT* non-randomised controlled trial

The initial database search generated 9587 studies. Once duplicates were removed, 6014 titles and abstracts were screened against the eligibility criteria. Of those, 5916 were excluded (“Eligibility Criteria” section). Following this, 98 titles were retrieved as full text and assessed for eligibility. Of those, 78 were excluded (Additional file 3: Table S3) with reasons for exclusion displayed in Fig. 2. On completion of these procedures, 20 studies were included for analysis in this systematic review.

### Study Characteristics

**Fig. 3:**
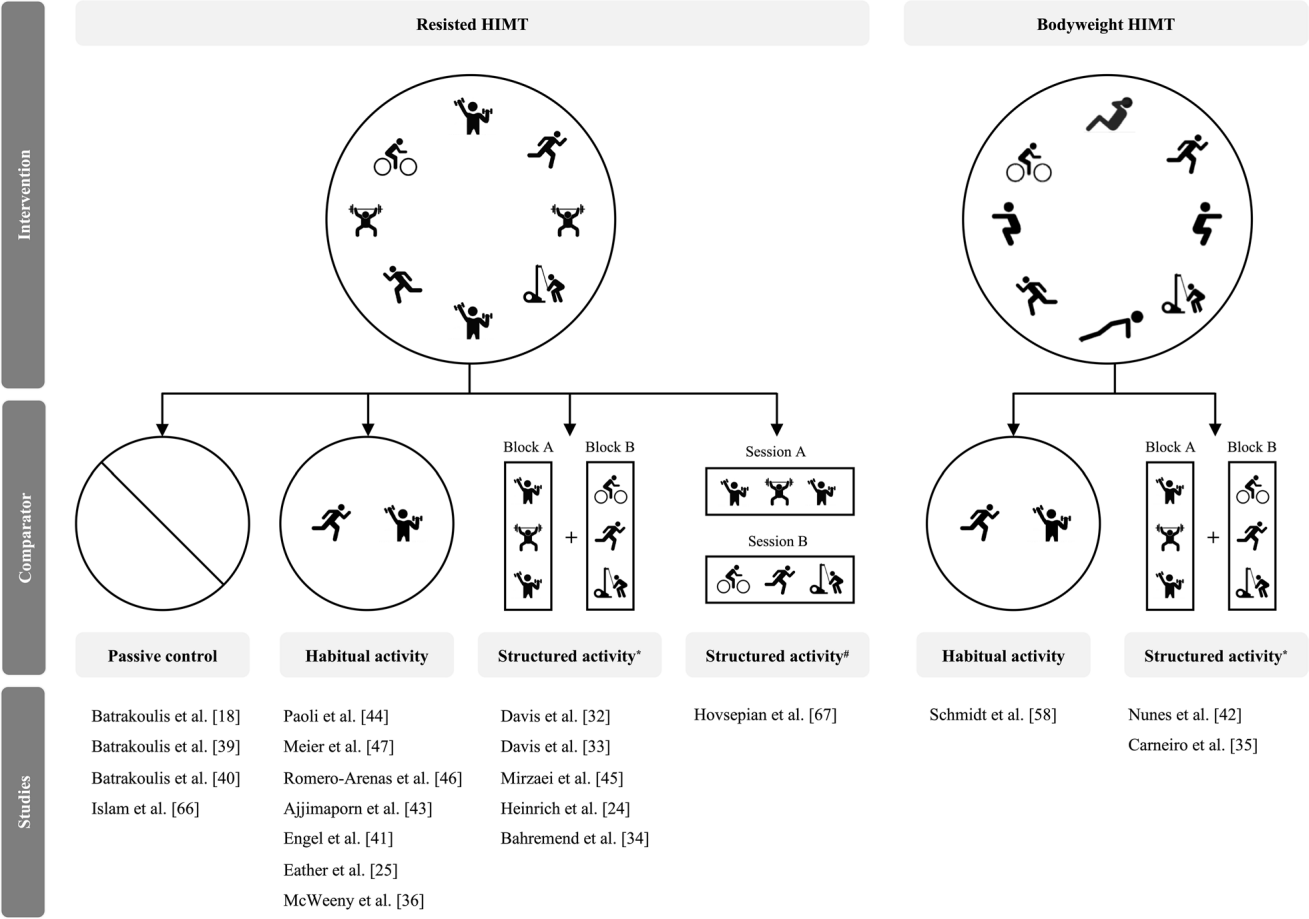
Intervention and comparator protocols of included studies. *HIMT* high-intensity multimodal training; * concurrent aerobic and resistance training distributed into a single session, # concurrent aerobic and resistance training distributed on different days

The 20 papers within this review examined 619 participants with mean age ranging from 18 to 63 (Additional file 2: Table S2). Three studies from the same authors observed the same group of participants [18, 39, 40]. Another two studies of the same group of authors reported on findings from the same subjects [32, 33]. Among the included studies, 4 compared HIMT to a passive control, 8 compared HIMT to a habitual activity control and 8 compared HIMT with structured activity (concurrent training) (Fig. 3). To meet the objectives of this review, the studies comparing HIMT to passive and habitual activity control groups will be discussed together. Studies that examined HIMT versus structured activity (concurrent training) will be discussed separately.

Within the studies comparing HIMT to passive or habitual activity controls, 6 measured aerobic fitness, 10 measured muscular fitness and 2 measured adherence-related subjective responses (Table 1). Among these studies, changes in muscular strength, endurance and power were examined by 8, 6 and 3 studies, respectively. Among the 8 studies comparing HIMT to structured activity (concurrent training), 3 measured aerobic fitness, 6 measured muscular fitness (5: strength, 2: endurance, 3: power) and only one study observed subjective responses (Table 1). HIMT interventions included high-intensity circuit bodyweight training, high-intensity circuit-type neuromuscular exercise training, high-intensity power training, Tabata, bodyweight HIIT, CrossFit® and integrated concurrent training. Passive and habitual activity control protocols involved no exercise and the continuation of regular activities, respectively. Structured activity (concurrent training) protocols included serial or blocked aerobic and resistance training in a single session (Fig. 1c) or on different days (Fig. 1d). The intervention and control group protocols of each study are displayed in Fig. 3. Further descriptive results of the included studies including publication details, participant characteristics, study methods, HIMT intervention, comparator and funding details are displayed in Additional file 2: Table S2. Pre- and post-intervention measures were extracted for primary (aerobic and muscular fitness) and secondary (subjective responses) outcome measures. Effect sizes (Hedges’ g) and their 95% confidence intervals were calculated for all outcome measures (“Data Analysis” section).

**Table 1:** Outcome measures observed by each included study

Reference	HIMT versus passive or habitual activity control												HIMT versus structured activity (concurrent training)							
	Paoli et al. [44]	Meier et al. [47]	Schmidt et al. [58]	Batrakoulis et al. [18]	Romero-Arenas t al. [46]	Ajjimaporn et al. [43]	Engel et al. [41]	Batrakoulis et al. [40]	Eather et al. [25]	Islam et al. [66]	McWeeny et al. [36]	Batrakoulis et al. [39]	Davis et al. [32]	Davis et al. [33]	Mirzaei et al. [45]	Heinrich et al. [24]	Carneiro et al. [35]	Nunes et al. [42]	Bahremand et al. [34]	Hovsepian et al. [67]
	*n* = 12												*n* = 8							
Aerobic Fitness	×		×	×		×	×			×			×						×	×
Muscular Fitness	×	×	×	×	×		×		×	×	×	×		×	×		×	×	×	×
Subjective Responses								×	×							×				

*HIMT* High-Intensity Multimodal Training

### Outcome Measures

**Fig. 4:**
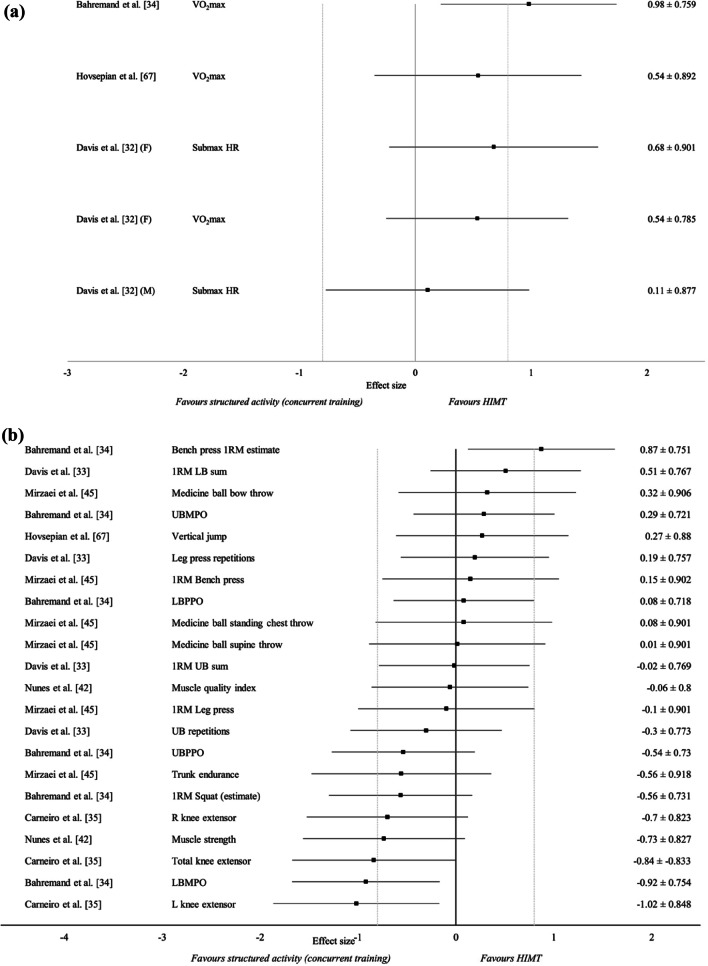
Effect sizes ± 95% confidence intervals of pre- to post-intervention between-group changes in **a** aerobic fitness for studies observing HIMT versus structured activity (concurrent training) and **b** muscular fitness for studies observing HIMT versus structured activity (concurrent training). *HIMT* high-intensity multimodal training, *1RM* 1 repetition maximum, *LB* lower body, *UB* upper body, *UBMPO* upper body mean power output, *LBPPO* lower body peak power output, *UBPPO* upper body peak power output, *LBMPO* lower body mean power output, *R* right, *L* left, *F* female, *M* male, *VO*_*2*_*max* maximal oxygen uptake, *HR* heart rate

Previous studies that compared HIMT to passive and habitual activity control groups generally demonstrated positive effects on aerobic fitness, muscular fitness and subjective responses in favour of the HIMT intervention. For detailed within- and between-group results of HIMT versus passive and habitual activity controls, see Additional file 4: Tables S4; Additional file 5: Table S5; Additional file 6: Table S6a, Fig. S1a–e. All studies comparing HIMT to structured activity (concurrent training) demonstrated a moderate to large effect on aerobic fitness in favour of the HIMT intervention (Table 2; Fig. 4a). Contrastingly, Davis et al. [32] did not demonstrate an effect (0.11 ± 0.877) in favour of either group on submaximal HR in male groups. Between-group effect sizes were unable to be calculated for female and male resting HR values and male VO_2_max data due to unreported data. Authors reported this was due to reduced sample size as a result of subject withdrawal and were contacted for data retrieval, without response. Another 4 studies demonstrated a small to large effect on muscular fitness measures in favour of the HIMT intervention versus structured activity (concurrent training). In contrast, 7 studies showed small to large effect in favour of the structured activity group. The 7 other studies observed a trivial effect on muscular fitness (Table 2; Fig. 4b). Exercise enjoyment was observed in one study only comparing HIMT to structured activity (concurrent training) [24]. Both training groups demonstrated increased exercise enjoyment, with a very large effect in favour of the HIMT group (2.71 ± 1.280) (Fig. S1f). For detailed within- and between-group results of HIMT versus structured activity (concurrent training), see Additional file 5: Table S5 and Additional file 6: Table S6b, Fig. S1f.

**Table 2:** Results of summary of within-group changes for studies observing HIMT versus structured activity (concurrent training)

Reference	Training group	Outcomes				
			Muscular fitness			
		Aerobic fitness	Muscular strength	Muscular endurance	Muscular power	Subjective responses
*HIMT versus structured activity (concurrent training)*						
Davis et al. [32]	Integrated CE (M)	↑^†^	×	×	×	×
	Integrated CE (F)	↑^†^ ↔	×	×	×	×
	Serial CE (M)	↔	×	×	×	×
	Serial CE (F)	↑ ↔	×	×	×	×
Davis et al. [33]	Integrated CE	×	↑^†^	↑^†^ ↔	×	×
	Serial CE	×	↑	↔	×	×
Mirzaei et al. [45]	Integrated CE	×	↑	↑	↑	×
	Serial CE	×	↑	↑	↔	×
Heinrich et al. [24]	CF	×	×	×	×	↑
	ART	×	×	×	×	↑^†^
Carneiro et al. [35]	HIBWT	×	↑	×	×	×
	COMT	×	↑^†^	×	×	×
Nunes et al. [42]	BW HIIT	×	↔	×	×	×
	ART	×	↑^†^	×	×	×
Bahremand et al. [34]	CF	↑	↑^†^	×	↑ ↔	×
	CT	↑	↑	×	↑ ↔	×
Hovespian et al. [67]	HIFT	↑	×	×	↔	×
	CSCT	↑	×	×	↔	×

*HIMT* high-intensity multimodal training, *CE* concurrent exercise, *CF* CrossFit®, *HICTBW* high-intensity circuit training with bodyweight, *COMT* combined training, *BWHIIT* bodyweight high-intensity interval training, *CT* combined training, *HIFT* high-intensity functional training, *CSCT* common strength and conditioning training, *M* male, *F* female, ↑ significant improvement, ↔ no significance change, ↓ significant decrease, † at least one significant difference compared to structured activity group, × not applicable

### Risk of Bias

The risk of bias assessment results, including sequence generation, allocation concealment, blinding, outcome data and selective reporting, are available in Table 3 and Additional file 7: Table S7. One study examined the effects of assignment to intervention for the intention to treat [25], and the remaining studies examined the effects of adherence to the intervention. Three studies from the same group of authors demonstrated random sequence generation and adequate allocation concealment [18, 39, 40]. Another two studies demonstrated random sequence generation without adequate allocation concealment [25, 41]. Nunes et al. [42] showed partial random sequence generation only. The remaining 14 studies demonstrated high risk of bias in inadequate randomisation and concealment procedures. Two studies by the same group of authors demonstrated concern in their randomisation and matching technique [32, 33]. This was highlighted by a discrepancy in the reporting of pairs. The remaining studies showed no significant differences at baseline or did not report this information. The same two studies demonstrated partial blinding of subjects [32, 33]. The participants were aware they were assigned to an intervention, but unaware of the aims. The remaining studies did not demonstrate adequate blinding of allocation to intervention or instructors delivering the intervention. Eight studies reported no or partially missing data [36, 41–47]. Other included studies either did not report on missing data or reasons for missingness. Three studies demonstrated partial blinding of testing assessors [32, 33, 46]. The other 17 studies showed inadequate blinding. All studies demonstrated moderate risk of bias for selective reporting. Included studies were unable to report adequate details of pre-registrations or lacked details of statistical methodologies if registration was provided. Overall, the included studies demonstrate high risk of bias particularly in the domains relating to deviation from intended interventions for the ‘per-protocol effect’, missing outcome data and outcome measurement.

**Table﻿ 3:** Quality assessment of included studies

Reference	Cochrane collaboration tool domain					
	Random sequence generation	Deviation from intended intervention	Missing outcome data	Outcome measurement	Selective reporting	Overall ROB
*HIMT versus passive or habitual activity control*						
Paoli et al. [44]	−	−	+	−	−	×
Meier et al. [47]	−	−	+	−	−	×
Schmidt et al. [58]	−	×	×	−	−	×
Batrakoulis et al. [18]	+	×	×	−	−	×
Romero-Arenas et al. [46]	−	−	+	+	−	×
Ajjimaporn et al. [43]	−	−	+	−	−	×
Engel et al. [41]	−	−	+	×	−	×
Batrakoulis et al. [40]	+	×	×	×	−	×
Eather et al. [25]^a^	−	+	×	×	−	×
Islam et al. [66]	−	×	×	−	−	×
McWeeny et al. [36]	−	−	+	−	−	×
Batrakoulis et al. [39]	+	×	×	−	−	×
*HIMT versus structured activity (concurrent training)*						
Davis et al. [32]	×	×	×	+	−	×
Davis et al. [33]	×	×	×	+	−	×
Mirzaei et al. [45]	−	−	+	−	−	×
Heinrich et al. [24]	−	×	×	×	−	×
Carneiro et al. [35]	−	×	×	−	−	×
Nunes et al. [42]	−	−	+	−	−	×
Bahremand et al. [34]	−	×	×	−	−	×
Hovsepian et al. [67]	−	×	×	−	−	×

*HIMT*, High-Intensity Multimodal Training; *ROB* risk of bias, + low risk of bias, × high risk of bias, − some risk of bias

^a^Intention-to-treat analysis

## Discussion

Despite HIMT providing a time-efficient alternative to traditional aerobic and resistance training modes, the exact magnitude of its effects on aerobic and muscular fitness remains unclear. While HIMT may be considered a novel term in the literature, it appears to be an attractive exercise mode in a real-world health and fitness setting [10]. Previous studies that have attempted to synthesise the literature demonstrate a lack of an operational term that broadly captures all combinations of aerobic and resistance training, e.g. HIFT, bodyweight HIIT and circuit HIIT [48–51]. These disparate styles of HIMT and unstandardised methods of reporting interventions make comparisons to other concurrent training modes difficult. For example, future implementation of checklists for reporting exercise interventions (e.g. Consensus on Exercise Reporting Template [CERT]) may improve consistency within the literature [52]. The primary aim of this systematic review was to examine the chronic or long-term effects of HIMT on aerobic and muscular fitness compared to other concurrent training modalities. A secondary aim of the review was to examine subjective responses in HIMT. This review also aimed to provide an operational definition of HIMT, to further capture the breadth of modalities existing within the literature and more effectively make comparisons to other methods of concurrent training.

### Summary of Evidence

Previous studies have attempted to examine the chronic effects of HIMT on numerous select outcomes in aerobic and muscular fitness and subjective responses. Mixed findings in these domains can be attributed to inconsistent definitions in the literature and methodological limitations. For example, studies have examined a variety of populations over a range of interventions varying in duration, volume, intensity and mode limiting the ability to comparatively examine their effects. Despite these inconsistencies, existing findings support positive implications for aerobic and muscular fitness. However, the magnitude of these effects seems to be related to the specific training principles (i.e. specificity, variation, progressive overload) of different HIMT styles. Given the emerging popularity of HIMT, a greater understanding of these effects is essential to guide future research in developing guidelines for practical implementation in real-world health and fitness settings.

### Aerobic Fitness

Previous studies have demonstrated moderate to large positive effects on aerobic fitness in favour of chronic HIMT participation compared to concurrent training or passive and habitual controls. Given the relationship between enhanced aerobic fitness and time spent at or near VO_2_max, these findings are likely attributed to the prescription of high-intensity workloads coupled with whole-body movement characteristics [11, 29]. HIMT sessions combine high-intensity bouts of gross whole-body movements and locomotor patterns that engage both upper and lower limbs. These components may provide a greater aerobic fitness stimulus than traditional resistance circuit training alone over a period of 4 weeks or longer [29, 48, 53]. For example, multi-station HIMT circuits and CrossFit® protocols comprised of whole-body exercises (e.g. step jack, wall-sit, wall push-up, sit-up hand reach and step-up onto aerobic step) have demonstrated improvements in VO_2_max, VO_2_peak and submaximal HR following HIMT participation [18, 34, 43]. Additional CrossFit® protocols comprised of whole-body aerobic and resistance-based movements have also been shown to acutely elicit high intensities of work (e.g. HR_max_ of > 180 bpm and blood lactate concentrations of 11–18 mmol/L) that compare to other forms of high-intensity endurance exercise [54, 55]. Despite popular high-intensity exercise protocols typically being shorter in session duration, this does not appear to limit aerobic fitness adaptations [56, 57]. For example, Schmidt et al. [58] demonstrated a large positive between-group effect on VO_2_max (1.15 ± 0.714) in favour of a training group (*n* = 15, females 20.5 ± 1.5 years) following eight weeks of single 14-min bodyweight circuit sessions three days/ week. Future studies should examine the effects of varied HIMT session styles (i.e. duration, volume, intensity and mode) on the magnitude of aerobic fitness adaptations to more clearly develop recommendations for practice in health and fitness settings for apparently healthy populations.

While previous findings demonstrate positive aerobic fitness adaptations following HIMT participation, the magnitude of these effects compared to other concurrent training modes remains unclear. Previous investigations demonstrate an inability to consistently prescribe, control and monitor exercise intensity and external work [e.g. objective (i.e. HR variables) vs. subjective measures (i.e. RPE)], reducing the ability to compare HIMT with other concurrent training modalities (i.e. match training dose). Additionally, heterogeneous intervention durations and work-to-rest ratios limit the understanding of the effects of chronic HIMT participation on aerobic fitness measures. A high risk of bias among included studies indicates that these findings should be examined with caution. More robust protocols that standardise reporting methods (i.e. duration, volume, intensity and mode) are required to allow the precise comparative effects of HIMT to be better understood. However, it is acknowledged that standardised exercise prescription and reporting in select styles of HIMT may increase internal validity yet decrease ecological validity (i.e. CrossFit®).

### Muscular Fitness

Previous research has demonstrated mixed outcomes of chronic HIMT participation on muscular fitness outcomes including strength, endurance and power, showing large effects in favour of both HIMT and other methods of concurrent training. Given the association of HIMT with external and/ or bodyweight resistance exercise, it is plausible that participation in HIMT may result in improvements in muscular fitness. Characteristic features of HIMT such as higher repetition ranges of whole-body movements performed in extended work bouts may increase time under tension, potentially providing a powerful musculoskeletal and neuromuscular stimulus that enhances muscular fitness adaptations [9, 39, 59]. For example, Batrakoulis et al. [39] demonstrated improvements in various 1RM lifts and measures of endurance after 40 weeks of up to 41-min sessions completed 3 days/week. Additionally, the fast-paced nature of HIMT may promote earlier recovery than other concurrent training methods, allowing for more rapid progressive overload and muscular fitness adaptations relative to the nature of the stimulus (i.e. duration, volume, intensity and mode) [60]. Davis et al. [60] demonstrated this concept in their observation of lower delayed-onset muscle soreness (i.e. rating of perceived pain) in an integrated concurrent aerobic and resistance training protocol compared to a serial concurrent protocol [60]. The authors proposed that aerobic elevation of HR preceding resistance exercise increases blood flow to the muscle and may stimulate long-term angiogenesis increasing the capillarisation rate of skeletal muscle [60]. This may reduce recovery periods, allowing participants to train more frequently and promote greater musculoskeletal adaptations.

HIMT seems to promote muscular fitness adaptations to an extent; however, select findings demonstrate reduced improvements compared to other concurrent training modes [35, 36, 42]. Select styles of HIMT (i.e. bodyweight HIIT) may involve lifting little or no load through higher repetition sets, which may contribute to lower magnitudes of musculoskeletal adaptations compared to traditional concurrent training [9]. For example, Nunes et al. [42] reported greater improvements in muscular strength (− 0.73 ± 0.827) and muscle quality index (muscle strength for the leg lean mass ratio) in the concurrent aerobic and resistance training group compared to the bodyweight only HIMT intervention. The authors attributed this outcome to greater load prescription of 70% 1RM in the concurrent training protocol compared to no resistance (bodyweight exercise) in the HIMT protocol. Similarly, Carneiro et al. [35] observed greater improvements in muscular strength in the concurrent training group (using loads of 70% 1RM) compared to the bodyweight HIMT group. Expectedly, these findings further indicate that external resistance can provide a more potent stimulus than bodyweight training alone [35, 42]. Future research should attempt to develop practical guidelines for different styles of HIMT (e.g. external resistance vs. bodyweight), whereby load prescription can more effectively prioritise specific muscular fitness adaptations (e.g. muscular strength vs. endurance).

Another possible explanation for attenuated musculoskeletal adaptations following chronic HIMT participation may be due to the ‘interference effect’. This phenomenon describes compromised muscular fitness gains when aerobic or resistance training blocks precede each other in a single session [61]. It has been suggested that HIIT-based concurrent training may diminish muscular strength gains but not hypertrophy when longer duration aerobic HIIT intervals are undertaken prior to resistance protocols [62]. This effect may be minimised with adequate rest periods or when aerobic training intervals are of higher intensity and shorter durations (e.g. repeat sprint training and sprint interval training) [62]. This concept may be relevant to HIMT, where improvements in muscular fitness may have been limited by alternate intervals of resistance and aerobic training among other variables of exercise prescription (i.e. sets, repetitions, rest period duration). For example, McWeeny et al. [36] demonstrated a very large between-group effect in (Wingate) lower body mean power (− 3.55 ± 1.406) in favour of the habitual activity control and no significant change in other measures of instantaneous muscular power (vertical jump, medicine ball toss) following a six-week HIMT intervention. Despite some findings suggesting that HIMT promotes musculoskeletal adaptations, the literature provides limited comparisons to structured concurrent training. Further well-controlled comparative studies that demonstrate greater homogeneity in intervention protocols are required to further understand the mechanisms of promoting and attenuating musculoskeletal adaptations in HIMT versus other concurrent training modes.

### Exercise Enjoyment and Other Adherence-Related Subjective Responses

Literature examining the chronic effects of HIMT participation on exercise enjoyment and other adherence-related attributes is limited. Three included studies measured subjective responses of interest to this review [24, 25, 40]. These studies demonstrated a range of positive effects on subjective responses in favour of HIMT including: exercise enjoyment, subjective vitality and introjected, intrinsic, identified and external regulation, psychological distress, HIIT self-efficacy and autonomous motivation [24, 25, 40]. While the reasons for exercise initiation and adherence are complex, these findings may assist in examining possible explanations for the popularity of HIMT [24–26, 40], namely the notion that HIMT may be enjoyable, which is based on HIIT being found to be more enjoyable than steady-state modalities in some populations [27, 28]. Given that the experience of enjoyment is suggested to impact exercise adherence by supporting intrinsic motivation, research should further investigate this concept in HIMT [6, 7]. The only study that observed exercise enjoyment demonstrated a large positive between-group effect in favour of the HIMT group (2.71 ± 1.280) [24]. Despite these findings, this study demonstrates a high risk of bias, specifically, in reported baselines differences in enjoyment between groups that were not adequately adjusted for in the analysis of the results. Moreover, the single-item scale used to measure exercise enjoyment in this study has been found to have fair test–retest reliability, suggesting that further development may be required [63]. While previous findings suggest HIMT may have a positive impact on subjective responses, limited research has attempted to identify and distinguish between the specific characteristics of HIMT that may promote greater exercise enjoyment and long-term exercise adherence. This may include the concurrent aerobic and resistance modality offering variety between sessions and suggested time-efficient endurance and strength adaptations [26]. Additional psychosocial factors that may mediate exercise enjoyment in HIMT (e.g. group training, instructor) have been shown to facilitate greater feelings of affiliation and social recognition [26, 40, 64, 65]. Further research is required to examine the components often associated with HIMT to better understand subjective responses to HIMT and assist in explaining the growing popularity of the training mode.

### Strengths and Limitations

This is the first systematic review to examine the effects of chronic HIMT participation on aerobic and muscular fitness and to compare various styles of HIMT to other structured concurrent training modalities, namely concurrent training where aerobic and resistance exercise are distributed into separate training blocks within a single session or on different days. Moreover, it is the first systematic review to examine enjoyability and other adherence-related subjective responses to various styles of HIMT. This may assist in efforts to better understand possible explanations for the growing uptake of HIMT as a time-efficient alternative to traditional concurrent training. This review provides an operational definition of HIMT, to effectively assess and compare between the various styles of HIMT that are broadly described in the literature. Heterogeneity of study methodologies and reported outcome measures did not allow for a meta-analysis to be performed. Limitations are present in the calculation of effect sizes and confidence intervals as some data were required to be manually extracted from the figures provided. A risk of bias may be present in the search term selection and exclusion criteria defined by authors. This may have resulted in potentially eligible studies being missed in the initial database search. For example, studies that did not clearly define or refer to the exercise intervention as ‘high’ intensity (i.e. used terms such as ‘vigorous’, ‘maximal effort’ and/ or ‘all-out effort’) but met the ACSM guidelines for high-intensity exercise may have been overlooked. However, reference lists and other resources were screened to achieve maximal literature saturation. Also, bias may exist in the included intervention and control protocols due to limited homogeneity of study protocols in the literature. To reduce this, the authors assessed studies for inclusion based on an original operational definition of HIMT. Additional bias may present in the inclusion and categorisation of selected outcome measures for reporting in this review. Only health- and fitness-related measures relevant to healthy populations were of interest, with other performance-related outcomes beyond the scope of this review. Furthermore, many studies did not report methods of prescribing, controlling or monitoring exercise intensity and used a small sample size of participants. An additional limitation is the high risk of bias demonstrated in most eligible studies. The heterogeneity of the experimental protocols and modality characterisation within the literature limits the ability to synthesise and compare the chronic effects of HIMT participation on aerobic and muscular fitness and exercise enjoyment and other adherence-related subjective responses. Additionally, there is reduced research comparing HIMT to concurrent training methods, limiting the understanding of the efficacy of HIMT versus traditional concurrent training methods. It should also be noted that the findings of this review may not be transferable to the general population, due to the diversity in participant demographics across the included studies. Finally, many of the included studies had no or limited funding. Improved funding may promote increased quality of experimental studies in HIMT.

### Future Research

Given the emerging popularity of HIMT, it is pertinent that research in this area is developed and critically assessed. Research suggests that HIMT has positive effects on aerobic fitness and mixed effects on musculoskeletal fitness. The magnitude of these effects remains unclear due to the lack of terminology that broadly captures all styles of aerobic and resistance training as well as heterogeneity of intervention protocols. Future studies should attempt to standardise reporting of training interventions (i.e. duration, volume, intensity and mode) against guidelines (e.g. CERT) and assess consistent chronic health and fitness outcomes. This may allow clearer comparisons of HIMT with other concurrent aerobic and resistance training protocols (i.e. matched training dose) and increase the understanding of its effects. However, standardisation of select HIMT protocols may increase internal validity, yet decrease ecological validity (i.e. CrossFit®). Additionally, these studies should endeavour to understand the mechanisms of the suggested aerobic and muscular fitness adaptations and more clearly examine possible interference effects of integrating aerobic and resistance training into a single exercise session. There is also a need to investigate feasible explanations for greater exercise enjoyment in HIMT. Future studies should attempt to examine the select features of HIMT that may mediate subjective responses. These findings may contribute to a more in depth understanding of exercise adherence in HIMT.

## Conclusion

HIMT participation demonstrates positive effects on aerobic fitness adaptations and mixed effects on muscular fitness outcomes including strength, endurance and power. However, these effects should not be overestimated due to heterogeneous experimental protocols and the inability to compare HIMT to other concurrent aerobic and resistance training methods. Furthermore, a greater understanding of the training behaviours and fitness trends associated with HIMT is required. While it is acknowledged that reasons for exercise engagement are complex, the affective response to HIMT (i.e. exercise enjoyment) should be highlighted, given the proposed association with greater exercise adherence. Further studies are required to understand this association to assist in explaining the growing popularity of the training mode. Finally, this review provided an operational definition of HIMT, to broadly conceptualise an emerging training mode and promote consistency in study protocols so more accurate conclusions and comparisons can be made.

## Supplementary Information

**Additional file 1.** Search strategy (29.03.2021).

**Additional file 2.** Descriptive results of included studies.

**Additional file 3.** List of excluded studies (n = 78).

**Additional file 4.** Results summary of within group changes for studies observing HIMT vs. passive or habitual activity control.

**Additional file 5.** Detailed results of included studies.

**Additional file 6.** Effect sizes ± 95% confidence intervals.

**Additional file 7.** Quality assessment of included studies.

## Data Availability

All data generated or analysed during this study are included in this document (and its electronic supplementary files).

## Abbreviations

HIMT: High-intensity multimodal training
RCT: Randomised controlled trial
ROB: Risk of bias
HIIT: High-intensity interval training
HIFT: High-intensity functional training
ACSM: American College of Sports Medicine
HRmax: Heart rate maximum
1RM: One repetition maximum
PRISMA: Preferred Reporting Items for Systematic Reviews and Meta-Analysis
PICOS: Population, Intervention, Comparator, Outcome, Study design
HIICT: High-intensity circuit training
MM-HIIT: Multimodal training high-intensity interval training
HIRT: High-intensity resistance training
CENTRAL: Cochrane Central Register of Controlled Trials
MeSH: Medical subject headings
SD: Standard deviation
HR: Heart rate
VO_2_max: Maximal oxygen uptake
VO_2_peak: Peak oxygen uptake
