# Supplementary material for: The Effects of High-Intensity Multimodal Training in Apparently Healthy Populations: A Systematic Review
Source: Sports Med Open. 2022 Mar 29;8:43. doi: 10.1186/s40798-022-00434-x (PMC8964907; doi:10.1186/s40798-022-00434-x)
Supplement: Supplementary file 1 — Additional file 1. Search strategy (29.03.2021). [file 40798_2022_434_MOESM1_ESM.docx]

**Electronic Supplementary Table S1** Search strategy (29.03.2021)

| **Database** | **Search strategy** | **Limits** | **Results** |
| --- | --- | --- | --- |
| **PubMed** | ((("multimodal training" OR "multi-modal training" OR "multimodal exercise" OR "multi-modal exercise" OR "functional training" OR "functional circuit training" OR "circuit training" OR "circuit resistance training" OR "resistance training" OR "strength training" OR "cardio-resistance training" OR "cardioresistance training" OR "exercise" OR "combined training" OR "combination training" OR "calisthenics" OR "bodyweight training" OR "body weight training" OR "body weight exercise" OR "bodyweight exercise" OR "total body exercise" OR "all extremity exercise" OR "high-velocity training" OR "high-velocity resistance training" OR "CrossFit" OR "crossfit" OR "interval training" OR "interval exercise" OR "impact exercise" OR "impact training" OR "functional movement*" OR "interval power training" OR "weightlift*" OR "olympic lift" OR “tabata” OR “suspension training”) AND ("strength" OR "resistance" OR "bodyweight" OR "body weight" OR "combined")) AND ("high-intensity" OR "high intensity" OR "vigorous" OR "vigorous intensity")) AND ("muscular fitness" OR "physical fitness" OR "endurance" OR "muscular endurance" OR "muscle endurance" OR "strength" OR "muscular strength" OR "muscle strength" OR "power" OR "muscular power" OR "muscle power" OR "peak power" OR "mean power" OR "1RM" OR "repetition max" OR "repetition maximum" OR "maximal repetitions" OR "strength test" OR "endurance test" OR "counter movement jump" OR "countermovement jump" OR "vertical jump" OR "aerobic capacity" OR "aerobic performance" OR "endurance" OR "aerobic endurance" OR "aerobic fitness" OR "cardiorespiratory fitness" OR "cardio-respiratory fitness" OR "cardio-vascular endurance" OR "cardiovascular endurance" OR "cardio-vascular fitness" OR "cardiovascular fitness" OR "VO2max" OR "VO2" OR "maximal oxygen uptake" OR "maximal test" OR "graded exercise test" OR "GXT" OR "submaximal exercise test" OR "heart rate maximum" OR "maximum heart rate" OR "HRmax" OR "max HR" OR "enjoy*" OR "adherence" OR "physical activity enjoyment" OR "perceptual response" OR "physical activity enjoyment scale" OR "enjoyment exercise scale" OR "affect" OR "affective valence" OR "affective state" OR "valence" OR "pleasur*" OR "intrinsic" OR "motivation" OR "self-efficacy") | N/A | **3.363** |
| **Web of Science** | (((("multimodal training" OR "multi-modal training" OR "multimodal exercise" OR "multi-modal exercise" OR "functional training" OR "functional circuit training" OR "circuit training" OR "circuit resistance training" OR "resistance training" OR "strength training" OR "cardio-resistance training" OR "cardioresistance training" OR "exercise" OR "combined training" OR "combination training" OR "calisthenics" OR "bodyweight training" OR "body weight training" OR "body weight exercise" OR "bodyweight exercise" OR "total body exercise" OR "all extremity exercise" OR "high-velocity training" OR "high-velocity resistance training" OR "CrossFit" OR "crossfit" OR "interval training" OR "interval exercise" OR "impact exercise" OR "impact training" OR "functional movement*" OR "interval power training" OR "weightlift*" OR "olympic lift" OR “tabata” OR “suspension training”) AND ("strength" OR "resistance" OR "bodyweight" OR "body weight" OR "combined")) AND ("high-intensity" OR "high intensity" OR "vigorous" OR "vigorous intensity")) AND ("muscular fitness" OR "physical fitness" OR "endurance" OR "muscular endurance" OR "muscle endurance" OR "strength" OR "muscular strength" OR "muscle strength" OR "power" OR "muscular power" OR "muscle power" OR "peak power" OR "mean power" OR "1RM" OR "repetition max" OR "repetition maximum" OR "maximal repetitions" OR "strength test" OR "endurance test" OR "counter movement jump" OR "countermovement jump" OR "vertical jump" OR "aerobic capacity" OR "aerobic performance" OR "endurance" OR "aerobic endurance" OR "aerobic fitness" OR "cardiorespiratory fitness" OR "cardio-respiratory fitness" OR "cardio-vascular endurance" OR "cardiovascular endurance" OR "cardio-vascular fitness" OR "cardiovascular fitness" OR "VO2max" OR "VO2" OR "maximal oxygen uptake" OR "maximal test" OR "graded exercise test" OR "GXT" OR "submaximal exercise test" OR "heart rate maximum" OR "maximum heart rate" OR "HRmax" OR "max HR" OR "enjoy*" OR "adherence" OR "physical activity enjoyment" OR "perceptual response" OR "physical activity enjoyment scale" OR "enjoyment exercise scale" OR "affect" OR "affective valence" OR "affective state" OR "valence" OR "pleasur*" OR "intrinsic" OR "motivation" OR "self-efficacy")) | N/A | **3,790** |
| **SPORTDiscus** | ((("multimodal training" OR "multi-modal training" OR "multimodal exercise" OR "multi-modal exercise" OR "functional training" OR "functional circuit training" OR "circuit training" OR "circuit resistance training" OR "resistance training" OR "strength training" OR "cardio-resistance training" OR "cardioresistance training" OR "exercise" OR "combined training" OR "combination training" OR "calisthenics" OR "bodyweight training" OR "body weight training" OR "body weight exercise" OR "bodyweight exercise" OR "total body exercise" OR "all extremity exercise" OR "high-velocity training" OR "high-velocity resistance training" OR "CrossFit" OR "crossfit" OR "interval training" OR "interval exercise" OR "impact exercise" OR "impact training" OR "functional movement*" OR "interval power training" OR "weightlift*" OR "olympic lift" OR “tabata” OR “suspension training”) AND ("strength" OR "resistance" OR "bodyweight" OR "body weight" OR "combined")) AND ("high-intensity" OR "high intensity" OR "vigorous" OR "vigorous intensity")) AND ("muscular fitness" OR "physical fitness" OR "endurance" OR "muscular endurance" OR "muscle endurance" OR "strength" OR "muscular strength" OR "muscle strength" OR "power" OR "muscular power" OR "muscle power" OR "peak power" OR "mean power" OR "1RM" OR "repetition max" OR "repetition maximum" OR "maximal repetitions" OR "strength test" OR "endurance test" OR "counter movement jump" OR "countermovement jump" OR "vertical jump" OR "aerobic capacity" OR "aerobic performance" OR "endurance" OR "aerobic endurance" OR "aerobic fitness" OR "cardiorespiratory fitness" OR "cardio-respiratory fitness" OR "cardio-vascular endurance" OR "cardiovascular endurance" OR "cardio-vascular fitness" OR "cardiovascular fitness" OR "VO2max" OR "VO2" OR "maximal oxygen uptake" OR "maximal test" OR "graded exercise test" OR "GXT" OR "submaximal exercise test" OR "heart rate maximum" OR "maximum heart rate" OR "HRmax" OR "max HR" OR "enjoy*" OR "adherence" OR "physical activity enjoyment" OR "perceptual response" OR "physical activity enjoyment scale" OR "enjoyment exercise scale" OR "affect" OR "affective valence" OR "affective state" OR "valence" OR "pleasur*" OR "intrinsic" OR "motivation" OR "self-efficacy") | N/A | **2,434** |

*N/A,* not applicable

**The Effects of High-Intensity Multimodal Training in Apparently Healthy Populations.**

**A Systematic Review.**

Sports Medicine - Open

Tijana Sharp^1^, Clementine Grandou^1^, Aaron J. Coutts^1^, Lee Wallace^1^

^1^Sport and Exercise Discipline Group, University of Technology, Human Performance Research Centre,

Moore Park, Sydney, Australia

Corresponding author: Tijana Sharp (tijana.sharp@uts.edu.au)
