# Supplementary material for: The Effects of High-Intensity Multimodal Training in Apparently Healthy Populations: A Systematic Review
Source: Sports Med Open. 2022 Mar 29;8:43. doi: 10.1186/s40798-022-00434-x (PMC8964907; doi:10.1186/s40798-022-00434-x)
Supplement: Supplementary file 2 — Additional file 2. Descriptive results of included studies. [file 40798_2022_434_MOESM2_ESM.docx]

**Electronic Supplementary Table S2a** Descriptive results of included studies comparing HIMT vs. passive or habitual activity control

| Reference | Year | Design | Participants | | | | HIMT intervention | | | | |
| --- | --- | --- | --- | --- | --- | --- | --- | --- | --- | --- | --- |
|  |  |  | No. | Sex | Age (mean±SD) | Baseline activity level | Duration (weeks) | Frequency (sessions/ week) | Volume | Intensity | Mode |
| Paoli | 2010 | RCT | 40 | M + F | 56 ± 2.7 | Not reported | 12 | 3 | 50 min total, AT: 3 min: 1 min intervals, RT: 3 sets x rest pause* with 60s rest, 1 set x 20 abdominal crunches | 65%: 75% estimated HRR, 6RM rest pause | Supervised alternate intervals of treadmill running and RT (lat pulldown, chest press, lateral shoulder raise, horizontal leg press, abdominal crunches). |
|  |  |  | CHG (n=10) |  | NR |  |  |  |  |  |  |
|  |  |  | CG (n=10)  CLG (n = 10 |  | NR  NR |  |  |  |  |  |  |
|  |  |  | EG (n=10) |  | HR |  |  |  |  |  |  |
|  |  |  |  |  |  |  |  |  |  |  |  |
| Meier | 2015 | RCT | 26 | M + F | 20.54 ± 1.17 | Regular PA, self-reported moderate to vigorous activity on 3-5 days/ week, >30min/day, total of 75-150 min/ week | 5 | 3 | WU: 5 min, HIIT: 20min (4 x 2 min set KB: 2 min set battle rope [15s work: 15s rest]), CD: 5 min | 8-10 RPE, KB 25% BW | HIIT using alternating KB exercises (regular swing, swing with goblet squat, shovel, 1-armed alternating swing, KB burpee) and battle ropes exercises (alternating waves, jumping jacks, parallel waves, rotational slam, jump slam). Supervision NR |
|  |  |  | HIIT (n=13) |  | NR |  |  |  |  |  |  |
|  |  |  | CG (n=13) |  | NR |  |  |  |  |  |  |
| Schmidt | 2016 | RCT | 96 |  |  | Recreationally active (participating in intramural sport or PA ≥ 30 mins for ≥ 2 but ≤ 3 days/ week | 8 | 3 | 1 x 7 min circuit, 30s effort, 10s active rest (to transition) | NR | 12 BW exercises (Jumping jacks, wall sit, push-up, abdominal crunch, step-up onto chair, squat, triceps dip on chair, plank, high knees/ running in place, lunge, push-up and rotation, side plank) performed in a circuit fashion supervised by student researcher |
|  |  |  | CT-7 (n=15) | M | 21.5 ± 1.5 |  |  |  |  |  |  |
|  |  |  | CT-7 (n=17) | F | 20.7 ± 1.3 |  |  |  |  |  |  |
|  |  |  | CT-14 (n=13) | M | 20.9 ± 0.95 |  | 8 | 3 | W1-4 as above, W4-8 two circuits performed consecutively | NR | 12 BW exercises (Jumping jacks, wall sit, push-up, abdominal crunch, step-up onto chair, squat, triceps dip on chair, plank, high knees/ running in place, lunge, push-up and rotation, side plank) performed in a circuit fashion supervised by student researcher |
|  |  |  | CT-14 (n=15) | F | 20.5 ± 1.5 |  |  |  |  |  |  |
|  |  |  | CG (n=15) | M | 21.4 ± 1.1 |  |  |  |  |  |  |
|  |  |  | CG (n=21) | F | 20.3 ± 1.1 |  |  |  |  |  |  |
| Batrakoulis | 2018 | RCT | 49 | F | 36.4 ± 4.4 | Inactive (<7,500 steps/ day, VO_2_max <30 ml/kg/min, accelerometry-based moderate-to- vigorous physical activity <30 min/day) | 40 | 3 | WU: 10 min, W1-7: 23 min total (2 rounds x 10 exercises, 20s work: 40s rest, 3min rest/ round), W8-14: 38 min total (3 rounds x 11 exercises, 30s work: 30s rest, 2.5 min rest/ round), W15-20: 41 min total (3 rounds x 12 exercises, 40s work: 20s rest, 2.5 min rest/ round), W21-40: 41 min total (3 rounds x 12 exercises, 40s work: 20s rest, 2 min rest/ round), CD: 5 min | ≥65% HRR, ‘as many reps as possible’. | WU: low-intensity endurance exercise, stretching, mobility, CINT: Supervised 10-12 whole body exercises performed in a circuit fashion. W1-7: over dome ankle touch, neutral grip row, sumo deadlift, straight-arm plank, low knee skip, bilateral wave, alternating static lunge, forearm plank, jumping jack, squat to overhead press. W8-14: straddle jump, wide grip row, sumo deadlift high pull, forearm plank, lateral shuffle, alternating wave, forward lunge with press, forearm plank with leg lift, split jack, lateral shuffle press, squat row. W15-20: split jack, Y deltoid raise, two arm swing, straight-arm reverse plank, heel flick, side-to-side wave, lunge to chest pass, shifting plank, ice skater, hockey slap shot, reverse fly with lunge, squat throw. W21-40: over dome hand touch, chest press, two-arm snatch, side plank rotation, high knee skip, slam, twisting chop, forearm plank with leg lift, burpee, axe chop, squat to overhead press, swing, CD: walking/ stretching |
|  |  |  | CINT TR (n = 14) |  | 36.4 ± 5.0 |  |  |  |  |  |  |
|  |  |  | CINT TRD (n = 14) |  | 36.9 ± 4.3 |  | 20 | 3 |  |  |  |
|  |  |  | CG (n = 21) |  | 36.0 ± 4.2 |  |  |  |  |  |  |
| Romero-Arenas | 2018 | RCT | 29 | M | 23.1 ± 2.7 | ≥ 6 months RT experience, ≥ 2 days/ week | 6 | 3 | 5-8 min total, 3-5 circuits (1 added every 2 weeks) of 10s work (5-10 reps): 15s rest (to transition), 90s rest between circuits | Maximum velocity, relative optimal individual load specific to exercise (~30% 1RM bench press, ~60% 1RM high pull, 0.075 kg/kg body mass braking resistance) | Alternate explosive RT (bench press, rebound jumps, high pull) and cycle ergometer Wingate effort performed in circuit fashion, supervised by Certified Strength and Conditioning Specialists. |
|  |  |  | HIPT (n = 10) |  | 22.7 ± 3.1 |  |  |  |  |  |  |
|  |  |  | TPT (n = 10) |  | 23.4 ± 1.9 |  |  |  |  |  |  |
|  |  |  | CG (n = 9) |  | 23.1 ± 2.5 |  |  |  |  |  |  |
| Ajjimaporn | 2019 | RCT | 24 | F | 35 ± 3 | <2 days/week or < 150 min exercise/ week, no RT experience | 4 | 3 | WU: 5 min, W1: 17.5 min total, 1 x 7.5 min circuit (30s work: 10s rest), W2-3: 35 min total, 2 x 7.5 min circuits (30s work: 10s rest) , W4: 52.5 min total, 3 x 7.5 min circuits (30s work: 10s rest), CD: 5 min | W1: 60% HRmax, W2-3: 66% HRmax, W4: 68% HRmax | HICTBW, 12 body weight exercises performed in a circuit fashion (step jack, wall-sit, wall push-up, sit-up hand reach, step-up onto aerobic step, half squat, triceps dip onto aerobic step, knee plank, hops, alternate lunges, plank with rotation, knee side plank). Supervision NR. |
|  |  |  | HICTBW (n = 12) |  | 35 ± 3 |  |  |  |  |  |  |
|  |  |  | CG (n = 12) |  | 35 ± 4 |  |  |  |  |  |  |
| Engel | 2019 | RCT | 20 | M + F | 36.2 ± 11.1 | Moderately trained, > 3 months continuous gym experience, 1-4 sessions/ week | 8 | 2 | Mean total duration of 30± 2 min, Tabata protocol (8 rounds x 20s work: 10s rest) | NR | Functional HIIT: TRX exercises and whole body exercises, squats, burpees, jumping jacks, chest press, mountain climbers, squats and rows, stand up/ lay down, high knees, push-ups, crunches. Guided by an experienced instructor |
|  |  |  | Functional HIIT (n = 10) |  | 35 ± 1.2 |  |  |  |  |  |  |
|  |  |  | CON (n = 10) |  | 37 ± 1.0 |  |  |  |  |  |  |
| Batrakoulis | 2020 | RCT | 49 | F | 36.4 ± 4.4 | Inactive (<7,500 steps/ day, VO_2_max <30 ml/kg/min, accelerometry-based moderate-to- vigorous physical activity <30 min/day) | 40 | 3 | Batrakoulis et al. 2018 | Batrakoulis et al. 2018 | Batrakoulis et al. 2018 |
|  |  |  | CINT TR (n = 14) |  | 36.4 ± 5.0 |  |  |  |  |  |  |
|  |  |  | CINT TRD (n = 14) |  | 36.9 ± 4.3 |  | 20 | 3 | Batrakoulis et al. 2018 | Batrakoulis et al. 2018 | Batrakoulis et al. 2018 |
|  |  |  | CG (n = 21) |  | 36.0 ± 4.2 |  |  |  |  |  |  |
| Eather | 2020 | RCT | 47 | F | 43.0 ± 10.7 | Self-identifying as sedentary (seated) for most of or all the time at work | 8 | 2-3 | WU: 2 min, 8min HIIT (W1-4: 30s work: 30s rest, W5-8: 40s work: 20s rest) | >85% HRmax | Combinations of aerobic (eg. shuttles skips, bear walks) exercise using either BW or basic equipment (eg. sports balls, 2-6kg medicine balls). Variety of HIIT workouts provided: eg. Office-HIIT, on the spot exercises eg. mountain climbers, squats, Work-HIIT, BW only, Sport-HIIT, basic sports equipment eg. basketball dribbling, medicine ball squat |
|  |  |  | Work-HIIT (n = 24) |  | NR |  |  |  |  |  |  |
|  |  |  | CG (n = 23) |  | NR |  |  |  |  |  |  |
|  |  |  |  |  |  |  |  |  |  |  |  |
| Islam | 2020 | RCT | 68 | M + F | 21 ± 3 | Self-reported <150 min/ week moderate to vigorous activity | 4 | 4 | WU: 2 min, Tabata: 4 min (20s work: 10s rest x 8 rounds) | "All-out", (females: 3-5 lb dumbbells, males 8-10 lbs dumbbells) | 1 of 4 different whole body exercises: burpee push-ups, mountain climber push-ups, jumping jacks, squat and thrust |
|  |  |  | Tabata (n = 26) |  | 21 ± 3 |  |  |  |  |  |  |
|  |  |  | VICT (n = 27) |  | 22 ± 4 |  |  |  |  |  |  |
|  |  |  | CTL (n = 15) |  | 21 ± 4 |  |  |  |  |  |  |
| McWeeny | 2020 | RCT | 30 | M + F | 23.2 ± 3.2 | Habitual PA (≥2 days/ week for ≥ 6 months), novice resistance trained, no current/ previous history of regular muscle strength or muscle endurance training | 6 | ≤ 4 | 12-20 min, varied sets and reps, no rest | As fast as possible with speed of movement emphasised | WU, strength/ skill component, main HIFT bout (deadlift, handstand push-ups, pull-ups, thrusters, push-ups, squats, kettlebell swings, box jumps, wall balls, burpees, hang cleans, front squats, back squats, standing shoulder press, triceps brachii dips, and overhead squats. |
|  |  |  | HIFT (n = 10) |  | 24.5 ± 1.0 |  |  |  |  |  |  |
|  |  |  | TRAD (n = 10) |  | 23.5 ± 1.1 |  |  |  |  |  |  |
|  |  |  | FE (n = 10) |  | 21.6 ± 0.8 |  |  |  |  |  |  |
| Batrakoulis | 2021 | RCT | 49 | F | 36.4 ± 4.4 | Inactive (<7,500 steps/ day, VO_2_max <30 ml/kg/min, accelerometry-based moderate-to- vigorous physical activity <30 min/day) | 40 | 3 | Batrakoulis et al. 2018 | Batrakoulis et al. 2018 | Batrakoulis et al. 2018 |
|  |  |  | CINT TR (n = 14) |  | 36.4 ± 5.0 |  |  |  |  |  |  |
|  |  |  | CINT TRD (n = 14) |  | 36.9 ± 4.3 |  | 20 | 3 | Batrakoulis et al. 2018 | Batrakoulis et al. 2018 | Batrakoulis et al. 2018 |
|  |  |  | CG (n = 21) |  | 36.0 ± 4.2 |  |  |  |  |  |  |

| Comparator group | | | | | Outcome measures | | |  |
| --- | --- | --- | --- | --- | --- | --- | --- | --- |
| Duration (weeks) | Frequency (sessions/ week) | Volume | Intensity | Mode | Aerobic Fitness | Muscular fitness (strength, endurance, power) | Subjective Responses | Sources of Funding |
| 12 | - | - | - | Passive control: no change to sedentary lifestyle | Submaximal HR | Strength (6RM bench press, 6RM leg press) | - | NR |
| 12 | 3 | 50 min total, WU: 5 min walking, AT: 8 min intervals, RT: 3 sets x 15RM with 60s rest, 1 set x 20 abdominal crunches after each resistance exercise, CD: 5 min walking + stretching | 65% HRmax, 15RM | Supervised alternate intervals of treadmill running and RT (underhand cable pulldowns, chest press, lateral shoulder raise, horizontal leg press, abdominal crunches) |  |  |  |  |
|  |  |  |  |  |  |  |  |  |
|  |  |  |  |  |  |  |  |  |
|  |  |  |  |  |  |  |  |  |
| 12 | 3 | 50 min total, WU: 5 min walking, AT: 30 min + 3 min each week, 4 sets x 20 abdominal crunches, CD: 5 min walking + stretching | 65% HRmax, 11-13 RPE | Supervised treadmill running, abdominal crunches |  |  |  |  |
| 5 | - | - | - | Habitual activity control: continued normal work out routines | - | Handgrip strength (R and L) | - | NR |
|  |  |  |  |  |  |  |  |  |
|  |  |  |  |  |  |  |  |  |
| 8 | - | - | - | Habitual activity control: maintained normal activity levels | VO_2_max | Strength (R handgrip, L handgrip), endurance (push-up) | - | NR |
|  |  |  |  |  |  |  |  |  |
|  |  |  |  |  |  |  |  |  |
|  |  |  |  |  |  |  |  |  |
|  |  |  |  |  |  |  |  |  |
|  |  |  |  |  |  |  |  |  |
|  |  |  |  |  |  |  |  |  |
| 40 | - | - | - | Passive control: no intervention | VO_2_max | 1RM leg press | - | Departmental funding |
|  |  |  |  |  |  |  |  |  |
|  |  |  |  |  |  |  |  |  |
|  |  |  |  |  |  |  |  |  |
|  |  |  |  |  |  |  |  |  |
|  |  |  |  |  |  |  |  |  |
|  |  |  |  |  |  |  |  |  |
|  |  |  |  |  |  |  |  |  |
| 6 | - | - | - | Habitual activity control: regular PA | - | Strength (1RM bench press, 1RM high pull), power (bench press, high pull, CMJ height, CMJ peak power, Wingate [Pmax, PmaxR, Pmean, PmeanR]) | - | Fundacio ́ n San Antonio (grant) |
|  |  |  |  |  |  |  |  |  |
| 6 | 3 | 18-30 min, 3-5 sets, 10s work (5-10 reps): 90s rest | As HIPT | Bench press, rebound jump, high pull, Wingate effort |  |  |  |  |
|  |  |  |  |  |  |  |  |  |
| 4 | - | - | - | Habitual activity control: continued usual activity | VO_2_peak (absolute), VO_2_ peak (relative), submaximal HR | - | - | No sources of funding |
|  |  |  |  |  |  |  |  |  |
|  |  |  |  |  |  |  |  |  |
|  |  |  |  |  |  |  |  |  |
| 8 | 1.5 ± 0.9 | - | - | Habitual activity control: maintained individual, not standardised training | HRmax | Strength (bourban test ventral, bourban test L, bourban test R), endurance (leg press, chest press, pulldown, back extension) | - | Deutsche Forschungsgemeinschaft, Baden-Württemberg Ministry of Science, Research, and the Arts and Ruprecht-Karls-Universität Heidelberg. |
|  |  |  |  |  |  |  |  |  |
|  |  |  |  |  |  |  |  |  |
| 40 | - | - | - | Passive control: no intervention | - | - | Psychological distress, subjective vitality, amotivation, external regulation, introjected regulation, intrinsic regulation, identified regulation | Departmental funding |
|  |  |  |  |  |  |  |  |  |
| 8 | - | - | - | Passive control: wait-list control: no intervention |  | Endurance (push-up), power (standing jump) | Autonomous motivation, HIIT self-efficacy | Australian Heart Foundation Innovation Grant |
|  |  |  |  |  |  |  |  |  |
|  |  |  |  |  |  |  |  |  |
|  |  |  |  |  |  |  |  |  |
| 4 | - | - | - | Habitual activity control: maintained current lifestyle habits | VO_2_peak | Endurance (back extension, push-up, sit-up, R plank, L plank) | - | NR |
|  |  |  |  |  |  |  |  |  |
| 4 | 4 | 30 min | 85% HRpeak | Treadmill running |  |  |  |  |
|  |  |  |  |  |  |  |  |  |
| 6 | - | - | - | Habitual activity control: free exercise, continued current PA habits | - | Strength (1RM, bench press, back squat, leg curl, leg extension, pull-up), endurance (BW squat, bent-arm hang, leg extension, bench press, leg curl), power (CMJ height, MB toss, [Wingate] LB PP, LB MP, UB PP, UB MP) | - | NR |
| 6 | - | Decreasing from 12-14 reps to 4-6 reps/ set each week | 9-10 RPE during last rep of each set | Flat bench press, incline chest fly, seated shoulder press, standing lateral shoulder raise, cable triceps brachii extensions, seated biceps brachii curls, leg press, squat, deadlift, prone hamstring curl, seated quadricep extension, seated calf press, and seated latissimus dorsi pull down. |  |  |  |  |
|  |  |  |  |  |  |  |  |  |
|  |  |  |  |  |  |  |  |  |
|  |  |  |  |  |  |  |  |  |
| 40 | - | - | - | Passive control: no intervention | - | Strength (1RM, chest press, lat pull-down, leg extension, leg curl), endurance (60s curl-up, 60s min push-up, 60s chair squat) | - | Departmental funding |
|  |  |  |  |  |  |  |  |  |
|  |  |  |  |  |  |  |  |  |
|  |  |  |  |  |  |  |  |  |

*RCT* randomised control trial, *HIMT* High-Intensity Multimodal Training, *CHG* circuit high-intensity group, *CLG* circuit low-intensity group, *EG* endurance group, *CG* control group, *HIIT* high-intensity interval training, *CT-7* 7 minute circuit training group, *CT-14* 14 minute circuit training group, *CINT* high-intensity circuit-type neuromuscular exercise training*, TR* 40 week training group, *TRD* 20 week training – 20 week de-training group, *HIPT* high-intensity power training, *TPT* traditional power training, *HICTBW* high-intensity circuit training using body weight, *CON* control group, *VICT* vigorous-intensity continuous training,  *CTL* control group, *HIFT* high-intensity functional training, *TRAD* traditional resistance training, *FE* free exercise, *F* female, *M* male, *PA* physical activity, *VO_2_max* maximal oxygen uptake, *RT* resistance training, *AT* aerobic training, * rest pause*, WU* warm up, *CD* cool down, *reps* repetitions, *KB* kettlebell, *W* week, *HRR* heart rate reserve, *1RM* 1 repetition maximum, *6RM* 6 repetition maximum, *RPE* rating of perceived exertion, *BW* body-weight, *NR* not reported, *HRmax* heart rate maximum, *TRX* total resistance exercise, *15RM* 15 repetition maximum, *HRpeak* heart rate peak, *VO_2_peak* peak oxygen uptake, *R* right, *L* left, *Pmax* maximum power, *PmaxR* relative maximum power, *Pmean* mean power, *PmeanR* relative mean power*, CMJ* counter movement jump, *MB* medicine ball, *LB* lower body, *UB* upper body, *PP* peak power, *MP* mean power

**Electronic Supplementary Table S2b** Descriptive results of included studies comparing HIMT vs. structured activity (concurrent training)

|  |  |  | Participants |  |  |  | HIMT Intervention | | | | |
| --- | --- | --- | --- | --- | --- | --- | --- | --- | --- | --- | --- |
| Reference | Year | Design | No. | Sex | Age (mean±SD) | Baseline activity level | Duration (weeks) | Frequency (sessions /week) | Volume | Intensity | Mode |
| Davis | 2008 | RCT | 48 | M + F |  | College athletes | 11 | 3 | Total 110min. WU: 20min, 75min integrated AT and RT (3 sets x 8-12 reps), CD: 15 min | AT: 60-84% HRR, RT: initial 50% 1RM (F)/ 65% 1RM (M), RPE regulated progression | Integrated concurrent AT and RT (seated inclined bilateral leg press, seated leg extension, seated leg curl, seated front lat pull-down, flat bench press, overhead press, biceps curl, triceps kickback, crunches) |
|  |  |  | Integrated CE M (n=10) |  | 20.36 ± 0.34 |  |  |  |  |  |  |
|  |  |  | Integrated CE F (n=14) |  | 19.7 ± 0.30 |  |  |  |  |  |  |
|  |  |  | Serial CE M (n=10) |  | 20.44 ± 0.41 |  |  |  |  |  |  |
|  |  |  | Serial CE F (n=14) |  | 19.4 ± 0.2 |  |  |  |  |  |  |
| Davis | 2008 | RCT | 28 | F |  | College athletes | 11 | 3 | Davis et al. 2008 | Davis et al. 2008 | Davis et al. 2008 |
|  |  |  | Integrated (n =14) |  | 19.7 ± 0.30 |  |  |  |  |  |  |
|  |  |  | Serial (n=14) |  | 19.4 ± 0.21 |  |  |  |  |  |  |
|  |  |  |  |  |  |  |  |  |  |  |  |
| Mirzaei | 2013 | RCT | 20 | M |  | Handball players, several-year-lasting sport experience | 9 | 3 | WU: 20 min, 75 min integrated AT and RT (W1-3: 3 sets x 10-12 reps, W4-9: 3 sets x 8-12 reps) exercise, CD: 15 min | RT: W1-3: 40% 1RM, W4-9: %1RM increased by ~5% biweekly. AT: 'vigorous'/ upper boundary of vigorous exercise, W1-2: 60-65% HRR, W3-4: 65-70% HRR, W5-6: 70-75% HRR, W7-9: 75-84% HRR | Integrated concurrent exercise : alternating sets of resistance exercise (leg press, knee flexion, knee extension, bench press, biceps curl, front lat pull-down, military press, crunches. 4s duty cycle [2s concentric, 2s eccentric]) with brief cardio-acceleration immediately prior to each set of resistance exercise |
|  |  |  | Integrated (n = 10) |  | 18.2 ± 0.27 |  |  |  |  |  |  |
|  |  |  | Serial (n = 10) |  | 18.1 ± 0.37 |  |  |  |  |  |  |
|  |  |  |  |  |  |  |  |  |  |  |  |
|  |  |  |  |  |  |  |  |  |  |  |  |
| Heinrich | 2014 | RCT | 23 | M + F | 26.8 ± 5.9 | Physically inactive | 8 | 3 | 60 min. WU: 10-15 min, instruction: 10-20min, WOD: 5-30min, CD: 5 min. Prescribed in singular or multiple combinations completed for time, reps or weight | Relative (self-selected) high-intensity | CF: aerobic (e.g. rowing), bodyweight (e.g. push-ups), and weightlifting (e.g. dead-lifts) exercises |
|  |  |  | CF (n = 12) |  |  |  |  |  |  |  |  |
|  |  |  | ART (n = 11) |  |  |  |  |  |  |  |  |
|  |  |  |  |  |  |  |  |  |  |  |  |
| Carneiro | 2018 | RCT | 25 | F |  | University women | 12 | 3 | WU: 5 min, 20min W1: 4 sets x 60s work: 4 min active recovery, W2: 6 sets x 60s work: 3 min active recovery, W3: 8 sets x 60s work: 2 min active recovery, W4-12: 10 sets x 60s work: 1 min active recovery, CD: 3 min | Work: 80-95% HRmax, 8-9 RPE, as fast and hard as possible. Recovery: <60% HRmax, <5 RPE. WU/ CD: <50% HRmax | Work: step ups (26cm step), body weight squats (90 degrees knee flexion). Recovery: light walking. WU/ CD: walking |
|  |  |  | HIBWT (n=10) |  | 24.3 ± 3.1 |  |  |  |  |  |  |
|  |  |  | COMT (n=15) |  | 25.8 ± 3.6 |  |  |  |  |  |  |
|  |  |  |  |  |  |  |  |  |  |  |  |
|  |  |  |  |  |  |  |  |  |  |  |  |
| Nunes | 2019 | RCT | 24 | F |  | No history of physical training practice > 1 year | 12 | 3 | WU: 5 min, HIIT: 28min, 4-10 rounds x (60s work (30s step climbing, 30s BW squats), 60s active recovery (light walk)). W1: 4 x 1 min: 4 x 4 min, W2: 6 x 1 min: 5 x 3 min, W3: 8 x 1 min: 8 x 2 min, W4-12: 1 0 x 1 min: 10 x 1 min | WU: 60% HRmax, Work: 80% of HRmax or RPE 7, "maximal reps". Active recovery: 60-70% HRmax or RPE 5 | HIIT: Step climbing (16cm), BW squats, recovery: light walking |
|  |  |  | HIIT (n = 12) |  | 63.0 (57.1-68.8) |  |  |  |  |  |  |
|  |  |  | CT (n = 12) |  | 62.9 (58.5-67.3) |  |  |  |  |  |  |
|  |  |  |  |  |  |  |  |  |  |  |  |
|  |  |  |  |  |  |  |  |  |  |  |  |
| Bahremand | 2020 | RCT | 30 | F |  | Regular PA in the last six months | 8 | 3 | Total 10-20min, varied prescription 'for time', AMRAP. No designated recovery periods | As quickly as possible at a high intensity (low repetition, high %1RM) | CF: resistance exercises (squat, press, deadlift, Olypmic lifts), traditional multi-joint, functional and basic gymnastic skills (handstands, ring and bar exercises) |
|  |  |  | CF (n = 16) |  | 30.8 ± 4.9 |  |  |  |  |  |  |
|  |  |  | CT (n = 14) |  | 30.9 ± 4.8 |  |  |  |  |  |  |
| Hovsepian | 2021 | RCT | 20 | F |  | Professional basketball players | 10 | 4 | 10-20min, 1-3 rounds of 8-10 minutes. Prescribed for time. No defined rest periods. | 70-90% HRmax, 60-70% 1RM, as fast as possible | CF: weightlifting (e.g. deadlift, squat, side lunge), gymnastics (e.g. burpee, pull-ups), metabolic conditioning (e.g. sprints, agility footwork) |
|  |  |  | HIFT (n = 10) |  | 23.5 ± 3.0 |  |  |  |  |  |  |
|  |  |  | CSCT (n = 10) |  | 21.0 ± 1.5 |  |  |  |  |  |  |

| Comparator | | | | | Outcome measures | | | Sources of Funding |
| --- | --- | --- | --- | --- | --- | --- | --- | --- |
| Duration (weeks) | Frequency (sessions/ week) | Volume | Intensity | Mode | Aerobic Fitness | Muscular Fitness (strength, endurance, power) | Subjective responses |  |
| 11 | 3 | Total 110 min. WU: 5 min, RT: 60 min (3 sets x 8-12 reps x 9 exercises), AT: 30min, CD: 15 min | AT: 60-84% HRR, RT: initial 50% 1RM (F)/ 65% 1RM (M), RPE regulated progression | Serial CE: RT (seated inclined bilateral leg press, seated leg extension, seated leg curl, seated fron lat pull-down, flat bench press, overhead press, biceps curl, triceps kickback, crunches), AT: vigorous treadmill running | RHR, active HR, estimated VO_2_max | - | - | This research was funded by the Division of Physical and Biological Sciences and the Office of Physical Education, Recreation, and Sports, of the University of California at Santa Cruz |
|  |  |  |  |  |  |  |  |  |
|  |  |  |  |  |  |  |  |  |
|  |  |  |  |  |  |  |  |  |
|  |  |  |  |  |  |  |  |  |
| 11 | 3 | Davis et al. 2008 | Davis et al. 2008 | Davis et al. 2008 | - | Strength (LB 1RM sum, UB 1RM sum), endurance (leg press reps , UB reps) | - | Davis et al. 2018_a_ |
|  |  |  |  |  |  |  |  |  |
|  |  |  |  |  |  |  |  |  |
|  |  |  |  |  |  |  |  |  |
| 9 | 3 | WU: 5 min, RT: 60 min (W1-3: 3 sets x 10-12 reps, 30-60s rest, W4-9: 3 sets x 8-12 reps, 30-60s rest), AT: 30min, CD: 15min | RT: W1-3: 40% 1RM, W4-9: %1RM increased by ~5% biweekly. AT: 'vigorous', W1-2: 60-65% HRR, W3-4: 65-70% HRR, W5-6: 70-75% HRR, W7-9: 75-84% HRR | RT: leg press, knee flexion, knee extension, bench press, biceps curl, front lat pull-down, military press, crunches. 4s duty cycle (2s concentric, 2s eccentric), AT: NR |  | Strength (1RM leg press, 1RM bench press), trunk endurance, power (2kg MB supine throw, 2kg MB standing chest throw, 2kg MB throw) | - | NR |
|  |  |  |  |  |  |  |  |  |
|  |  |  |  |  |  |  |  |  |
|  |  |  |  |  |  |  |  |  |
|  |  |  |  |  |  |  |  |  |
| 8 | 3 | AT : 50 min, RT : 20 min on Mon and Wed, 3 sets x (W2–3 : 15 reps, W4–5 : 12 reps, W6–7 : 10 reps, W8 : 8 reps) + 3 sets x 15 crunches, 60s rest | AT : W1-4 :40-50% HRR, W5-8 50-60% HRR, RT : W2–3 : 50% 1RM, W4–5 : 60% 1RM, W6–7 : 70% 1RM, W8 : 75% 1RM | RT : full body resistance exercises (biceps curls, military press, lat pulldown, leg extension, triceps pulldown, bench press, reverse leg curl, seated leg press), AT : Continuous aerobic activity on various machines | - | - | Exercise enjoyment | Kansas State University Academic Excellence Fund |
|  |  |  |  |  |  |  |  |  |
|  |  |  |  |  |  |  |  |  |
|  |  |  |  |  |  |  |  |  |
| 12 | 3 | Total 60min. AT: 30 min, RT: 3 sets x 8-12 reps x 5 exercises, 90s rest between sets and exercises. W1: AT: 15min, RT: + 1 set x 8-12 reps x 5 exercises, 90s rest. W2: AT: 20 min, RT: 2 sets x 8-12 reps, 90s rest. W3: AT: 25min, RT: 2 sets x 8-12 reps, 90s rest. W4-12: AT: 12-30min, RT: 3 sets x 8-12 reps, 90s rest. | AT: 70% HRmax, 5-6 RPE, RT: 70% 1RM | Walking, total body resistance exercises (45 degree half squat, bench press, leg curl, rowing machine, unilateral leg extension) | - | R knee extensor, L knee extensor, total knee extensor | - | Funda ̧cão de Amparo à Pesquisa do Estado de Minas Gerais—FAPEMIG and by Coordena ̧cão de Aperfei ̧coamento de Pessoal de Nível Superior—CAPES. |
|  |  |  |  |  |  |  |  |  |
|  |  |  |  |  |  |  |  |  |
|  |  |  |  |  |  |  |  |  |
|  |  |  |  |  |  |  |  |  |
| 12 | 3 | Total 60 min: AT: Up to 30min, RT:5 exercises x 3 sets x 8-12 reps, 90s rest between sets and exercises (W1: AT: 15min, RT: 1 set, W2: AT: 20min, RT: 2 sets, W3: AT: 25min, RT: 2 sets, W4-12: AT: 30min, RT: 3 sets) | Walk: 70% HRmax or RPE 5-6, RT : 70% 1RM | RT: 45 degree half squat (smith machine), bench press, leg curl, rowing machine, unilateral leg extension, AT : Walking | - | 1RM unilateral leg press, muscle quality index | - | Partially funded by the Coordenac ̧a ̃o de Aperfeic ̧oamento de Pessoal de N ́ıvel Superior – Brasil (CAPES, Code 001) and Fundac ̧a ̃o de Amparo e Pesquisa de Minas Gerais (FAPEMIG). |
|  |  |  |  |  |  |  |  |  |
|  |  |  |  |  |  |  |  |  |
|  |  |  |  |  |  |  |  |  |
|  |  |  |  |  |  |  |  |  |
| 8 | 3 | Total 22.5min. Volume NR | W1: 60% HRmax/ 60% 1RM W2: 65% HRmax/ 65% 1RM, W3: 65% HRmax/ 65% 1RM, W4: 70% HRmax/ 70% 1RM, W5: 70% HRmax, 70% 1RM, W6: 75% HRmax/ 75% 1RM, W7: 75% HRmax/ 75% 1RM, W8: 80% HRmax/ 80% 1RM (1RM testing assessed every 2 weeks) | AT (running), followed by RT. | VO_2_max | Strength (1RM bench press estimate, 1RM squat estimate), power (Wingate [LBPPO, LBMPO, UBPPO, UBMPO]) | - | No funding |
|  |  |  |  |  |  |  |  |  |
|  |  |  |  |  |  |  |  |  |
| 10 | 4 | RT session: 75-90min, 2-3 sets x 8-15 reps, AT session: 20-30min, varied drill length and rest periods | RT: 75-85% 1RM, AT: NR | RT : abdominal work, squat or leg press, bench press or DB incline press, machine row or lat pull-down, reverse hyperextension, DB bench press leg extension, single-leg squat, DB leg curl, crossover side step-up, swiss ball chin-up, heel raise, Conditioning : Jump rope routine, ladder sprints, agility drills. | VO_2_max | Vertical jump | - | NR |
|  |  |  |  |  |  |  |  |  |
|  |  |  |  |  |  |  |  |  |
|  |  |  |  |  |  |  |  |  |

*RCT* randomised control trial, *HIMT* High-Intensity Multimodal Training, *CE* concurrent exercise, *M* male, *F* female *CF* CrossFit^®^, *ART* aerobic and resistance training, *HIBWT* high-intensity body-weight training, *COMT* combined training, *BW HIIT* body weight high-intensity interval training, *HIIT* high-intensity interval training, *CT* combined training, *CSCT* common strength and conditioning training, *PA* physical activity, *WU* warm up, *CD* cool down, *RT* resistance training, *AT* aerobic training, *W* week, *WOD* workout of the day, *BW* body-weight, *AMRAP* as many rounds as possible, *HRR* heart rate reserve, *1RM* 1 repetition maximum, *RPE* rating of perceived exertion, *HRmax* heart rate maximum, *NR* not reported, *RHR* resting heart rate, *HR* heart rate, *VO_2_max* maximal oxygen uptake, *LB* lower body, *UB* upper body, *reps* repetitions, *MB* medicine ball, *R* right, *L* left, *LBPPO* lower body peak power output, *LBMPO* lower body mean power output, *UBPPO* upper body peak power output, *UBMPO* upper body mean power output

**The Effects of High-Intensity Multimodal Training in Apparently Healthy Populations.**

**A Systematic Review.**

Sports Medicine - Open

Tijana Sharp^1^, Clementine Grandou^1^, Aaron J. Coutts^1^, Lee Wallace^1^

^1^Sport and Exercise Discipline Group, University of Technology, Human Performance Research Centre, Moore Park, Sydney, Australia

Corresponding author: Tijana Sharp (tijana.sharp@uts.edu.au)
