# Supplementary material for: The Effects of High-Intensity Multimodal Training in Apparently Healthy Populations: A Systematic Review
Source: Sports Med Open. 2022 Mar 29;8:43. doi: 10.1186/s40798-022-00434-x (PMC8964907; doi:10.1186/s40798-022-00434-x)
Supplement: Supplementary file 3 — Additional file 3. List of excluded studies (n = 78). [file 40798_2022_434_MOESM3_ESM.docx]

**Electronic Supplementary Table S3** List of excluded studies (n = 78)

| **Exclusion criteria** | **Reference** |
| --- | --- |
|  |  |
| **Wrong intervention (n = 27)** | Abe [1]; Amaro-Gehete [2]; Arciero [3]; Brunelli [4]; DelVecchio [5]; Dornemann [6]; Fyfe [7]; Hagerman [8]; Hermassi [9]; Hollerbach [10]; Holviala [11]; Hurley [12]; Jones [13]; Kalapotharakos [14]; Karavita [15]; Lee [16]; Locke [17]; Mamen[18] ; McCarthy [19]; Moro [20]; Ouerghi [21]; Prasertsri [22]; Ramirez-Velez [23]; Reljic [24]; Sculthorpe [25]; Sousa [26]; Werner [27] |
| **NRCT (n =21)** | Azeem [28]; Azeem [29]; Azeem [30]; Bellew [31]; Chin [32]; Cho [33]; Chtara [34]; Cordina [35]; Cosgrove [36]; Fajrin [37]; Falatic [38]; Greenlee [39]; Henry [40]; Ikarugi [41]; Karabulut [42]; Kemmler [43]; Kemmler [44]; Khan [45]; Kokorev [46]; McRae [47]; Wanna [48] |
| **Wrong comparator (n = 9)** | Brown [49]; Buckley [50]; Chukhlantseva [51]; Fincher [52]; Schaun [53]; Schaun [54]; Sobrero [55]; Sperlich [56]; Wilke [57] |
| **Wrong study design (n = 7)** | Brosmer [58]; Denadai [59]; Garcia [60]; Grant [61]; Poudevigne [62]; Takakura [63]; Wescott [64] |
| **Wrong population (n = 6)** | Alonzo-Fernandes [65] ; Bento [66]; Garcia-Pinillos [67]; Jiminez-Garcia [68]; Martins [69]; Rosendahl [70] |
| **Conference abstract (n = 5)** | DuManior [71] ; Fincher [72]; Green [73]; Kalapotharkos [74]; Potteiger [75] |
| **Preregistration (n = 3)** | Batrakoulis [76]; Frimpong [77]; Ramirez-Velez [78] |

**References:**

1. Abe T, DeHoyos DV, Pollock ML, Garzarella L. Time course for strength and muscle thickness changes following upper and lower body resistance training in men and women. Eur J Appl Physiol. 2000;81(3):174-80.

2. Amaro-Gahete FJ, De-la-O A, Jurado-Fasoli L, Martinez-Tellez B, Ruiz JR, Castillo MJ. Exercise training as a treatment for cardiometabolic risk in sedentary adults: are physical activity guidelines the best way to improve cardiometabolic health? The fit-ageing randomized controlled trial. J Clin Med. 2019;8(12):2097.

3. Arciero PJ, Gentile CL, Martin-Pressman R, Ormsbee MJ, Everett M, Zwicky L, et al. Increased dietary protein and combined high intensity aerobic and resistance exercise improves body fat distribution and cardiovascular risk factors. Int J Sport Nutr Exerc Metab. 2006;16(4):373-92.

4. Brunelli DT, Chacon-Mikahil MP, Gáspari AF, Lopes WA, Bonganha V, Bonfante IL, et al. Combined training reduces subclinical inflammation in obese middle-age men. Med Sci Sports Exerc. 2015;47(10):2207-15.

5. Del Vecchio L, Stanton R, Reaburn P, Macgregor C, Meerkin J, Villegas J, et al. Effects of combined strength and sprint training on lean mass, strength, power, and sprint performance in masters road cyclists. J Strength Cond Res. 2019;33(1):66-79.

6. Dornemann TM, McMurray RG, Renner JB, Anderson JJ. Effects of high-intensity resistance exercise on bone mineral density and muscle strength of 40-50-year-old women. J Sports Med Phys Fitness. 1997;37(4):246-51.

7. Fyfe JJ, Bartlett JD, Hanson ED, Stepto NK, Bishop DJ. Endurance training intensity does not mediate interference to maximal lower-body strength gain during short-term concurrent training. Front Physiol. 2016;7:487.

8. Hagerman FC, Walsh SJ, Staron RS, Hikida RS, Gilders RM, Murray TF, et al. Effects of high-intensity resistance training on untrained older men. i. strength, cardiovascular, and metabolic responses. J Gerontol A Biol. 2000;55(7):B336-B46.

9. Hermassi S, Chelly MS, Fieseler G, Bartels T, Schulze S, Delank KS, et al. Short-term effects of combined high-intensity strength and sprint interval training on anthropometric characteristics and physical performance of elite team handball players. Sportverletz Sportschaden. 2017;31(4):231-9.

10. Hollerbach BS, Jahnke SA, Poston WSC, Harms CA, Heinrich KM. Examining a novel firefighter exercise training program on simulated fire ground test performance, cardiorespiratory endurance, and strength: a pilot investigation. J Occup Med Toxicol. 2019;14(1):12.

11. Holviala J, Kraemer WJ, Sillanpää E, Karppinen H, Avela J, Kauhanen A, et al. Effects of strength, endurance and combined training on muscle strength, walking speed and dynamic balance in aging men. Eur J Appl Physiol. 2012;112(4):1335-47.

12. Hurley BF, Seals DR, Ehsani AA, Cartier LJ, Dalsky GP, Hagberg JM, et al. Effects of high-intensity strength training on cardiovascular function. Med Sci Sports Exerc. 1984;16(5):483-8.

13. Jones TW, Howatson G, Russell M, French DN. Performance and neuromuscular adaptations following differing ratios of concurrent strength and endurance training. J Strength Cond Res. 2013;27(12):3342-51.

14. Kalapotharakos VI, Michalopoulou M, Godolias G, Tokmakidis SP, Malliou PV, Gourgoulis V. The effects of high- and moderate-resistance training on muscle function in the elderly. J Aging Phys Act. 2004;12(2):131-43.

15. Karavirta L, Häkkinen A, Sillanpää E, García-López D, Kauhanen A, Haapasaari A, et al. Effects of combined endurance and strength training on muscle strength, power and hypertrophy in 40–67-year-old men. Scand J Med Sci Sports. 2011;21(3):402-11.

16. Lee MG, Park KS, Kim DU, Choi SM, Kim HJ. Effects of high-intensity exercise training on body composition, abdominal fat loss, and cardiorespiratory fitness in middle-aged Korean females. Appl Physiol Nutr Metab. 2012;37(6):1019-27.

17. Locke SR, Bourne JE, Beauchamp MR, Little JP, Barry J, Singer J, et al. High-intensity interval or continuous moderate exercise: a 24-week pilot trial. Med Sci Sports Exerc. 2018;50(10):2067-75.

18. Mamen A, Fredriksen, PM. Tyre-pulling as concurrent training. Sport Sci Health. 2019;15(1):49-58.

19. McCarthy JP, Pozniak MA, Agre JC. Neuromuscular adaptations to concurrent strength and endurance training. Med Sci Sports Exerc. 2002;34(3):511-9.

20. Moro T, Tinsley G, Bianco A, Gottardi A, Gottardi GB, Faggian D, et al. High intensity interval resistance training (HIIRT) in older adults: Effects on body composition, strength, anabolic hormones and blood lipids. Exp Gerontol. 2017;98:91-8.

21. Ouerghi N, Fradj MKB, Bezrati I, Khammassi M, Feki M, Kaabachi N, et al. Effects of high-intensity interval training on body composition, aerobic and anaerobic performance and plasma lipids in overweight/obese and normal-weight young men. Biol Sport. 2017;34(4):385-92.

22. Prasertsri P, Padkao T. Efficacy of high-intensity interval resistance training on pulmonary function and respiratory muscle strength in university athletes. J Exerc Physiol Online. 2021;24(1):93-105.

23. Ramírez-Vélez R, Castro-Astudillo K, Correa-Bautista JE, González-Ruíz K, Izquierdo M, García-Hermoso A, et al. The effect of 12 weeks of different exercise training modalities or nutritional guidance on cardiometabolic risk factors, vascular parameters, and physical fitness in overweight adults: cardiometabolic high-intensity interval training-resistance training randomized controlled study. J Strength Cond Res. 2020;34(8):2178-2188.

24. Reljic D, Frenk F, Herrmann HJ, Neurath MF, Zopf Y. Effects of very low volume high intensity versus moderate intensity interval training in obese metabolic syndrome patients: a randomized controlled study. Sci Rep. 2021;11(1):2836.

25. Sculthorpe N, Herbert P, Grace FM. Low-frequency high-intensity interval training is an effective method to improve muscle power in lifelong sedentary aging men: a randomized controlled trial. J Am Geriatr Soc. 2015;63(11):2412-3.

26. Sousa AC, Neiva HP, Gil MH, Izquierdo M, Rodríguez-Rosell D, Marques MC, et al. Concurrent training and detraining: the influence of different aerobic intensities. J Strength Cond Res. 2020;34(9):2565-74.

27. Werner TJ, Pellinger TK, Rosette VD, Ortlip AT. Effects of a 12-week resistance training program on arterial stiffness: a randomized controlled trial. J Strength Cond Res. 2019.

28. Azeem K, Tabur E. Influence of low, medium and high intensity of resistance training on muscular hypertrophy, and selected health related fitness variables among underweight males. Int J Appl Exerc Physiol. 2017;6(4):14-25

29. Azeem K. Impact of low, medium and high intensity of resistance training on some selected fitness variables and muscular hypertrophy among underweight males. Ovidius Univ Ann Ser Phys Educ Sport Sci Mov Health Online. 2018:18(2):154.

30. Azeem K, Ameer AA. Resistance training with low to high intensity protocol on muscular hypertrophy, and selected health related fitness variables among overweight males. Int J Appl Exerc Physiol. 2019;8(4):108-14.

31. Bellew JW. The effect of strength training on control of force in older men and women. Aging Clin Exp Res. 2002;14(1):35-41.

32. Chin EC, Yu AP, Lai CW, Fong DY, Chan DK, Wong SH, et al. Low-frequency hiit improves body composition and aerobic capacity in overweight men. Med Sci Sports Exerc. 2020;52(1):55-56.

33. Cho J-K, Lee S-H, Lee J-Y, Kang H-S. Randomized controlled trial of training intensity in adiposity. Int J Sports Med. 2011;32(6):468-75.

34. Chtara M, Chaouachi A, Levin GT, Chaouachi M, Chamari K, Amri M, et al. Effect of concurrent endurance and circuit resistance training sequence on muscular strength and power development. The J Strength Cond Res. 2008;22(4):1037-45.

35. Cordina RL, O'Meagher S, Karmali A, Rae CL, Liess C, Kemp GJ, et al. Resistance training improves cardiac output, exercise capacity and tolerance to positive airway pressure in Fontan physiology. Int J Cardiol. 2013;168(2):780-8.

36. Cosgrove SJ, Crawford DA, Heinrich KM. Multiple fitness improvements found after 6-months of high intensity functional training. Sports (Basel). 2019;7(9):203.

37. Fajrin F, Kusnanik NW, Wijono. Effects of high intensity interval training on increasing explosive power, speed, and agility. J Phys Conf Ser. 2018;947(1).

38. Falatic JA, Plato PA, Holder C, Finch D, Han K, Cisar CJ. Effects of kettlebell training on aerobic capacity. J Strength Cond Res. 2015;29(7):1943-7.

39. Greenlee TA, Greene DR, Ward NJ, Reeser GE, Allen CM, Baumgartner NW, et al. Effectiveness of a 16-week high-intensity cardioresistance training program in adults. J Strength Cond Res. 2017;31(9):2528-41.

40. Henry RN, Anshel M, Michael T. Effects of aerobic and circuit training on fitness and body image among women. J Sport Behav. 2006;29:281-303.

41. Ikarugi H, Shibata M, Shibata S, Ishii H, Taka T, Yamamoto J. High intensity exercise enhances platelet reactivity to shear stress and coagulation during and after exercise. Pathophysiol Haemost Thromb. 2003;33(3):127-33.

42. Karabulut M, Bemben DA, Sherk VD, Anderson MA, Abe T, Bemben MG. Effects of high-intensity resistance training and low-intensity resistance training with vascular restriction on bone markers in older men. Eur J Appl Physiol. 2011;111(8):1659-67.

43. Kemmler W, von Stengel S, Weineck J, Lauber D, Kalender W, Engelke K. Exercise effects on menopausal risk factors of early postmenopausal women: 3-yr erlangen fitness osteoporosis prevention study results. Med Sci Sports Exerc. 2005;37(2):194-203.

44. Kemmler W, Engelke K, von Stengel S, Weineck J, Lauber D, Kalender WA. Long-term four-year exercise has a positive effect on menopausal risk factors: the erlangen fitness osteoporosis prevention study. J Strength Cond Res. 2007;21(1):232-9.

45. Khan W, Arif T, Muhammad K, Sohail SN, Kriventsova IV. Effects of varied packages of plyometric training on selected motor ability components among university students. Phys educ stud. 2020;24(5):278-85.

46. Kokorev DA, Vyprikov DV, Bodrov IM. Adapted version of multisport crossfit system for academic physical education service. Theory and Practice of Physical Culture. 2018(8).

47. McRae G, Payne A, Zelt JG, Scribbans TD, Jung ME, Little JP, et al. Extremely low volume, whole-body aerobic–resistance training improves aerobic fitness and muscular endurance in females. Appl Physiol Nutr Metab. 2012;37(6):1124-31.

48. Wanna S, Mitranun W. Comparison of HIIT and HIPT on oxygen consumption.J Exerc Physiol Online. 2021;24(1):7-17.

49. Brown EC, Hew-Butler T, Marks CRC, Butcher SJ, Choi MD. The impact of different high-intensity interval training protocols on body composition and physical fitness in healthy young adult females. Biores Open Access. 2018;7(1):177-85.

50. Buckley S, Knapp K, Lackie A, Lewry C, Horvey K, Benko C, et al. Multimodal high-intensity interval training increases muscle function and metabolic performance in females. Appl Physiol Nutr Metab. 2015;40(11)1157-62.

51. Chukhlantseva N, Cherednychenko I, Kemkina V. The influence of high-intensity functional training versus resistance training on the main physical fitness indicators in women aged 25-35 years. Trends Sport Sci. 2020;27(3):157-65.

52. Fincher GE. The effect of high intensity strength training on anaerobic power and endurance. Ann Arbor Michigan. 1996.

53. Schaun GZ, Pinto SS, Brasil B, Nunes GN, Alberton CL. Neuromuscular adaptations to sixteen weeks of whole-body high-intensity interval training compared to ergometer-based interval and continuous training. J Sport Sci. 2019;37(14):1561-9.

54. Schaun GZ, Alberton CL. Using bodyweight as resistance can be a promising avenue to promote interval training: enjoyment comparisons to treadmill-based protocols. Res Q Exerc Sport. 2020:1-9.

55. Sobrero G, Schafer M, Tolbert TA, Crandall, J, Brown J, Esslinger T, et al. A comparison of high intensity functional training and circuit training on health and performance variables in women: a pilot study. Women Sport Phys Act J. 2017;25(1):1-10.

56. Sperlich B, Wallmann-Sperlich B, Zinner C, Von Stauffenberg V, Losert H, Holmberg HC. Functional high-intensity circuit training improves body composition, peak oxygen uptake, strength, and alters certain dimensions of quality of life in overweight women. Front Physiol. 2017;8:172.

57. Wilke J, Kaiser S, Niederer D, Kalo K, Engeroff T, Morath C, et al. Effects of high-intensity functional circuit training on motor function and sport motivation in healthy, inactive adults. Scan J Med Sci Sports. 2019;29(1):144-53.

58. Brosmer R. High intensity weight training for individualized physical fitness. National Strength & Conditioning Association Journal. 1985;7(1):58-9.

59. Denadai BS, Greco CC. Resistance training and exercise tolerance during high-intensity exercise: moving beyond just running economy and muscle strength. J Appl Physiol. 2018;124(2):526-8.

60. Garcia M, Custodio E. Home quarantine - based rhythmic exercises: new fitness assessment and intervention in teaching physical education. Phys educ stud. 2021;25(1).

61. Grant CC, Mongwe L, Janse van Rensburg DC, Fletcher L, Wood PS, Terblanche E, et al. The difference between exercise-induced autonomic and fitness changes measured after 12 and 20 weeks of medium-to-high intensity military training. J Strength Cond Res. 2016;30(9):2453-9.

62. Poudevigne M, Day C, Campbell E, Mills D, Poter R, Zornosa X, et al. Fit for Fire: A 10-week low-cost hift experiential learning initiative between underrepresented kinesiology undergraduates and hypertensive deconditioned firefighters improves their health and fitness. Educ Sci. 2021;11(1):33.

63. Takakura R, Masayoshi K, Tsubota Y. The effects of a short term high-intensity circuit training exercise in university students. Int J Physiother. 2015;2(4):602-9.

64. Wescott WL. Benefits of high-intensity strength training. American Fitness. 1996;14(4):24-5.

65. Alonso-Fernández D, Lima-Correa F, Gutierrez-Sánchez A, De Vicuña, OAG. Effects of a high-intensity interval training protocol based on functional exercises on performance and body composition in handball female players. J Hum Sport Exerc. 2017;12(4):1186-98.

66. Bento P, Rodacki A. Muscle function in aged women in response to a water-based exercises program and progressive resistance training. Geriatr Gerontol Int. 2014;15(11)1193-200.

67. García-Pinillos F, Laredo-Aguilera JA, Muñoz-Jiménez M, Latorre-Roman PA. Effects of 12-week concurrent high-intensity interval strength and endurance training program on physical performance in healthy older people. J Strength Cond Res. 2019;33(5):1445-52.

68. Jiménez-García JD, Martínez-Amat A, De la Torre-Cruz MJ, Fábrega-Cuadros R, Cruz-Díaz D, Aibar-Almazán A, et al. Suspension training HIIT improves gait speed, strength and quality of life in older adults. Int J Sports Med. 2019;40(02):116-24.

69. Martins FM, de Paula Souza A, Nunes PRP, Michelin MA, Murta EFC, Resende EAMR, et al. High-intensity body weight training is comparable to combined training in changes in muscle mass, physical performance, inflammatory markers and metabolic health in postmenopausal women at high risk for type 2 diabetes mellitus: A randomized controlled clinical trial. Exp Gerontol. 2018;107:108-15.

70. Rosendahl E, Lindelöf N, Littbrand H, Yifter-Lindgren E, Lundin-Olsson L, Håglin L, et al. High-intensity functional exercise program and protein-enriched energy supplement for older persons dependent in activities of daily living: a randomised controlled trial. Aust J Physiother. 2006;52(2):105-13.

71. DuManoir G, Haykowsky M, Taylor D, Syrotuik D, Bell G. Combined strength and endurance training does not improve left ventricular systolic function in response to a high-intensity exercise in females. Faseb J. 2014;28(1).

72. Fincher GE. The effect of high intensity resistance training on sustained anaerobic power output among collegiate football players. Med Sci Sports Exerc. 2001;33(5).

73. Green ES, Williams ER, McCully KK, Jenkins NT. Enhanced strength, power, work capacity, and fatigue resistance in high intensity functional training athletes. Faseb J. 2019;33(S1):695.11.

74. Kalapotharakos V, Michalopoulos M, Vassilakou F, Tokmakidis S, Malliou V, Beneka A. High intensity resistance training improves strength and functional performance in elderly. (Abstract). European College of Sport Science, Proceedings of the 7th annual congress of the European College of Sport Science, Athens. 2002:1019.

75. Potteiger JA, Thyfault JP, Carper MJ, Hulver MW. A comparison of high intensity resistance training versus endurance training on cardiovascular disease risk factors in overweight males. Faseb J. 2002;16(4):A94.

76. Batrakoulis A, Fatouros IG, Chatzinikolaou A, Draganidis D, Georgakouli K, Papanikolaou K, et al. Dose-response effects of high-intensity interval neuromuscular exercise training on weight loss, performance, health and quality of life in inactive obese adults: Study rationale, design and methods of the DoIT trial. Contemp Clin Trials Commun. 2019;15:100386.

77. Frimpong E, Dafkin C, Donaldson J, Millen AME, Meiring RM. The effect of home-based low-volume, high-intensity interval training on cardiorespiratory fitness, body composition and cardiometabolic health in women of normal body mass and those with overweight or obesity: protocol for a randomized controlled trial. BMC Sports Sci Med Rehabil. 2019;11(1):39.

78. Ramírez-Vélez R, Hernandez A, Castro K, Tordecilla-Sanders A, González-Ruíz K, Correa-Bautista JE, et al. High intensity interval- vs resistance or combined- training for improving cardiometabolic health in overweight adults (cardiometabolic HIIT-RT study): study protocol for a randomised controlled trial. Trials. 2016;17(1):298.

**The Effects of High-Intensity Multimodal Training in Apparently Healthy Populations.**

**A Systematic Review.**

Sports Medicine - Open

Tijana Sharp^1^, Clementine Grandou^1^, Aaron J. Coutts^1^, Lee Wallace^1^

^1^Sport and Exercise Discipline Group, University of Technology, Human Performance Research Centre,

Moore Park, Sydney, Australia

Corresponding author: Tijana Sharp (tijana.sharp@uts.edu.au
