# Supplementary material for: The Effects of High-Intensity Multimodal Training in Apparently Healthy Populations: A Systematic Review
Source: Sports Med Open. 2022 Mar 29;8:43. doi: 10.1186/s40798-022-00434-x (PMC8964907; doi:10.1186/s40798-022-00434-x)
Supplement: Supplementary file 4 — Additional file 4. Results summary of within group changes for studies observing HIMT vs. passive or habitual activity control. [file 40798_2022_434_MOESM4_ESM.docx]

**Electronic Supplementary Table S4** Results summary of within group changes for studies observing HIMT vs. passive or habitual activity control

| Reference | Training group | Outcomes | | | | |  |  |
| --- | --- | --- | --- | --- | --- | --- | --- | --- |
|  |  | Aerobic fitness | Muscular strength | Muscular endurance | Muscular power | Subjective Responses |  |  |
|  |  |  | Muscular fitness | | |  |  |  |
|  |  |  | Muscular strength | Muscular endurance | Muscular power |  |  |  |
|  |  |  |  |  |  |  |  |  |
|  |  |  |  |  |  |  |  |  |
|  |  |  |  |  |  |  |  |  |
|  |  |  |  |  |  |  |  |  |
| HIMT vs. passive or habitual activity control | | | |  |  |  |  |  |
| Paoli et al. [44] | CHG | ↑* | ↑* | x | x | x |  |  |
|  | CG (habitual activity) | ↔ | ↔ | x | x | x |  |  |
|  |  |  |  |  |  |  |  |  |
| Meier et al. [47] | HIIT | x | ↑↔ | x | x | x |  |  |
|  | CG (habitual activity) | x | ↔ | x | x | x |  |  |
|  |  |  |  |  |  |  |  |  |
| Schmidt et al. [58] | CT-7 (M) | ↔ | ↑↔ | ↑ | x | x |  |  |
|  | CT-7 (F) | ↔ | ↔ | ↑ | x | x |  |  |
|  | CT-14 (M) | ↔ | ↑↔ | ↑ | x | x |  |  |
|  | CT-14 (F) | ↑ | ↔ | ↑ | x | x |  |  |
|  | CG (habitual activity M) | ↔ | ↔ | ↔ | x | x |  |  |
|  | CG (habitual activity F) | ↔ | ↔ | ↔ | x | x |  |  |
|  |  |  |  |  |  |  |  |  |
| Batrakoulis et al. [18] | CINT (TR) | ↑* | ↑ | x | x | x |  |  |
|  | CINT (TRD) | ↑* | ↑ | x | x | x |  |  |
|  | CG (passive) | ↓ | ↔ | x | x | x |  |  |
|  |  |  |  |  |  |  |  |  |
| Romero-Arenas et al. [46] | HIPT | x | ↑*↔ | x | ↑*↔ | x |  |  |
|  | CG (habitual activity) | x | ↔ | x | ↔ | x |  |  |
|  |  |  |  |  |  |  |  |  |
| Ajjimaporn et al. [43] | HICTBW | ↑* | x | x | x | x |  |  |
|  | CG (habitual activity) | ↔ | x | x | x | x |  |  |
|  |  |  |  |  |  |  |  |  |
| Engel et al. [41] | Functional HIIT | ↔ | ↑*↔ | ↑*↔ | x | x |  |  |
|  | CON (habitual activity) | ↔ | ↔ | ↔ | x | x |  |  |
|  |  |  |  |  |  |  |  |  |
| Batrakoulis et al. [40] | CINT (TR) | x | x | x | x | ↑*↔ |  |  |
|  | CINT (TRD) | x | x | x | x | ↑*↔* |  |  |
|  | CG (passive) | x | x | x | x | ↔ |  |  |
|  |  |  |  |  |  |  |  |  |
| Eather et al. [25] | HIIT | x | x | ↑* | ↑* | ↑* |  |  |
|  | CG (passive) | x | x | ↔ | ↔ | ↔ |  |  |
|  |  |  |  |  |  |  |  |  |
| Islam et al. [66] | Tabata | ↔ | x | ↑↔ | x | x |  |  |
|  | CTL (habitual activity) | ↔ | x | ↔ | x | x |  |  |
|  |  |  |  |  |  |  |  |  |
| McWeeny et al. [36] | HIFT | x | ↑ | ↑↔* | ↓*↔ | x |  |  |
|  | Free exercise (habitual activity) | x | ↑↔ | ↑↔ | ↑↔↓ | x |  |  |
|  |  |  |  |  |  |  |  |  |
| Batrakoulis et al. [39] | CINT (TR) | x | ↑* | ↑* | x | x |  |  |
|  | CINT (TRD) | x | ↑* | ↑* | x | x |  |  |
|  | CG (passive) | x | ↔ | ↔ | x | x |  |  |

*HIMT* High-Intensity Multimodal Training, *CHG* circuit high-intensity group, *HIIT* High-Intensity Interval Training, *CG* control group, *CT-7* 7 minute circuit training group, *CT-14* 14 minute circuit training group, *F* female, *M* male, *CINT* high-intensity circuit-type neuromuscular exercise training*, TR* 40 week training group, *TRD* 20 week training – 20 week de-training group, *HIPT* High-Intensity power training, *HICTBW* high-intensity circuit training using body weight, *HIFT* High-Intensity Functional Training, ↑ significant improvement, ↔ no significance change, ↓ significant decrease, * at least one significant change compared to non-structured exercise group, *x* not applicable

**The Effects of High-Intensity Multimodal Training in Apparently Healthy Populations.**

**A Systematic Review.**

Sports Medicine - Open

Tijana Sharp^1^, Clementine Grandou^1^, Aaron J. Coutts^1^, Lee Wallace^1^

^1^Sport and Exercise Discipline Group, University of Technology, Human Performance Research Centre,

Moore Park, Sydney, Australia

Corresponding author: Tijana Sharp (tijana.sharp@uts.edu.au)
