# Supplementary material for: The Effects of High-Intensity Multimodal Training in Apparently Healthy Populations: A Systematic Review
Source: Sports Med Open. 2022 Mar 29;8:43. doi: 10.1186/s40798-022-00434-x (PMC8964907; doi:10.1186/s40798-022-00434-x)
Supplement: Supplementary file 5 — Additional file 5. Detailed results of included studies. [file 40798_2022_434_MOESM5_ESM.docx]

**Electronic Supplementary Table S5a** Detailed results of included studies comparing HIMT vs. passive or habitual activity control

| Reference | Training group | Aerobic Fitness | Muscular Fitness | | | Subjective Responses | | | | | | | |  |  |
| --- | --- | --- | --- | --- | --- | --- | --- | --- | --- | --- | --- | --- | --- | --- | --- |
|  |  |  | Muscular Strength | Muscular Endurance | Muscular Power | Exercise enjoyment | Psychological distress | Subjective vitality | Amotivation | External regulation | Introjected regulation | Intrinsic regulation | Identified regulation | HIIT self-efficacy | Autonomous motivation |
| Paoli et al. [44] | CHG | ↓ Submax HR* | ↑ 6RM bench press*, ↑ 6RM leg press* |  |  |  |  |  |  |  |  |  |  |  |  |
|  | CG (habitual activity) | ↔ Submax HR | ↔ 6RM bench press, ↔ 6RM leg press |  |  |  |  |  |  |  |  |  |  |  |  |
| Meier et al. [47] | HIIT |  | ↑ R grip strength, ↔ L grip strength |  |  |  |  |  |  |  |  |  |  |  |  |
|  | CG (habitual activity) |  | ↔ R grip strength, ↔ L grip strength |  |  |  |  |  |  |  |  |  |  |  |  |
| Schmidt  et al. [58] | CT-7 (M) | ↔ VO_2_max | ↔ R grip strength, ↑ L grip strength | ↑ Push-up |  |  |  |  |  |  |  |  |  |  |  |
|  | CT-7 (F) | ↔ VO_2_max | ↔ R grip strength, ↔ L grip strength | ↑ Push-up |  |  |  |  |  |  |  |  |  |  |  |
|  | CT-14 (M) | ↔ VO_2_max | ↑ R grip strength, ↔ L grip strength | ↑ Push-up |  |  |  |  |  |  |  |  |  |  |  |
|  | CT-14 (F) | ↑ VO_2_max | ↔ R grip strength, ↔ L grip strength | ↑ Push-up |  |  |  |  |  |  |  |  |  |  |  |
|  | CG (habitual activity M) | ↔ VO_2_max | ↔ R grip strength, ↔ L grip strength | ↔ Push-up |  |  |  |  |  |  |  |  |  |  |  |
|  | CG (habitual activity F) | ↔ VO_2_max | ↔ R grip strength, ↔ L grip strength | ↔ Push-up |  |  |  |  |  |  |  |  |  |  |  |
| Batrakoulis  et al. [18] | CINT (TR) | ↑ VO_2_max * | ↑ 1 RM leg press |  |  |  |  |  |  |  |  |  |  |  |  |
|  | CINT (TRD) | ↑ VO_2_max * | ↑ 1 RM leg press |  |  |  |  |  |  |  |  |  |  |  |  |
|  | CG (passive) | ↓ VO_2_max | ↔ 1RM leg press |  |  |  |  |  |  |  |  |  |  |  |  |
| Romero-Arenas  et al. [46] | HIPT |  | ↔ 1RM bench press, ↑ 1RM high pull* |  | ↔ PP bench press, ↑PP high pull*, ↑ CMJ height*, ↔ CMJ peak power, Wingate (↑ Pmax*, ↑ PmaxR*, ↑ Pmean*, ↑ PmeanR*) |  |  |  |  |  |  |  |  |  |  |
|  |  |  |  |  |  |  |  |  |  |  |  |  |  |  |  |
|  | CG (habitual activity) |  | ↔ 1RM bench press, ↔ 1RM high pull |  | ↔ Bench press, ↔ high pull, ↔ CMJ height, ↔ CMJ peak power, Wingate (↔ Pmax, ↔ PmaxR, ↔ Pmean, ↔ PmeanR) |  |  |  |  |  |  |  |  |  |  |
|  |  |  |  |  |  |  |  |  |  |  |  |  |  |  |  |
| Ajjimaporn et al. [43] | HICTBW | ↑ VO_2_peak* (absolute), ↑ VO_2_peak* (relative), ↓ submaximal HR* |  |  |  |  |  |  |  |  |  |  |  |  |  |
|  |  |  |  |  |  |  |  |  |  |  |  |  |  |  |  |
|  | CG (habitual activity) | ↔ VO_2_peak (absolute), ↔ VO_2_peak (relative), ↔ submaximal HR |  |  |  |  |  |  |  |  |  |  |  |  |  |
|  |  |  |  |  |  |  |  |  |  |  |  |  |  |  |  |
| Engel et al. [41] | Functional HIIT | ↔ HRmax | Bourban test (↔ ventral, ↔ R, ↑ L*) | ↔ Leg press, ↑ chest press*, ↑ pulldown*, ↔ back extension |  |  |  |  |  |  |  |  |  |  |  |
|  | CON (habitual activity) | ↔ HRmax | Bourban test (↔ ventral, ↔ R, ↔ L) | ↔ Leg press, ↔ chest press, ↔ pulldown, ↔ back extension |  |  |  |  |  |  |  |  |  |  |  |
| Batrakoulis  et al. [40] | CINT (TR) |  |  |  |  |  | ↓* | ↑* | ↔ | ↓* | ↑ | ↑* | ↑* |  |  |
|  | CINT (TRD) |  |  |  |  |  | ↓* | ↑* | ↔ | ↔ | ↔ | ↑ | ↔* |  |  |
|  | CG (passive) |  |  |  |  |  | ↔ | ↔ | ↔ | ↔ | ↔ | ↔ | ↔ |  |  |
| Eather et al. [25] | HIIT |  |  | ↑ Push-up* | ↑ Standing jump* |  |  |  |  |  |  |  |  | ↑* | ↑* |
|  | CG (passive) |  |  | ↔ Push-up | ↔ Standing jump |  |  |  |  |  |  |  |  | ↔ | ↔ |
| Islam et al. [66] | Tabata | ↔ VO_2_peak |  | ↔ Back extension, ↑ push-up, ↔sit-up, ↑ R plank, ↔ L plank |  |  |  |  |  |  |  |  |  |  |  |
|  | CTL (habitual PA) | ↔ VO_2_peak |  | ↔ Back extension, ↔ push-up, ↔sit-up, ↔ R plank, ↔ L plank |  |  |  |  |  |  |  |  |  |  |  |
| McWeeny  et al. [36] | HIFT |  | ↑ 1RM back squat, ↑ 1RM bench press, ↑ 1RM leg extension, ↑1RM pull-up, ↑ 1RM leg curl | ↑ BW squat, ↑ bent arm hang, ↔ leg ext*, ↔ bench press, ↔ leg curl | ↔ CMJ height, ↔ MB toss, Wingate (↔ LB PP, ↓ LB MP*, ↔ UB PP, ↔ UB MP) |  |  |  |  |  |  |  |  |  |  |
|  |  |  |  |  |  |  |  |  |  |  |  |  |  |  |  |
|  | FE |  | ↔ 1RM back squat, ↑ 1RM bench press, ↑1RM leg extension, ↑ 1RM pull-up, ↑ 1RM leg curl | ↑ BW squat, ↑ bent arm hang, ↔ leg ext, ↔ bench press, ↔ leg curl | ↑ CMJ height, ↑ MB toss, Wingate (↓ LB PP, ↔ LB MP, ↔ UB PP, ↔ UB MP) |  |  |  |  |  |  |  |  |  |  |
|  |  |  |  |  |  |  |  |  |  |  |  |  |  |  |  |
| Batrakoulis  et al. [39] | CINT (TR) |  | ↑ 1RM chest press*, ↑ 1RM lat pull down*, ↑ 1RM leg extension*, ↑ 1RM leg curl* | 60s (↑ curl-up*, ↑ chair squat*, ↑ push-up*) |  |  |  |  |  |  |  |  |  |  |  |
|  | CINT (TRD) |  | ↑ 1RM chest press*, ↑ 1RM lat pull down*, ↑ 1RM leg extension*, ↑ 1RM leg curl* | 60s (↑ curl-up*, ↑ chair squat*, ↑ push-up*) |  |  |  |  |  |  |  |  |  |  |  |
|  | CG (passive) |  | ↔ 1RM chest press, ↔ 1RM lat pull down, ↔ 1RM leg extension, ↔ 1RM leg curl | 60s (↔ curl-up, ↔ chair squat, ↔ push-up) |  |  |  |  |  |  |  |  |  |  |  |

*RCT* randomised control trial, *HIMT* High-Intensity Multimodal Training, *CHG* circuit high-intensity group, *CG* control group, *HIIT* High-Intensity Interval Training, *CT-7* 7 minute circuit training group, *CT-14* 14 minute circuit training group, *F* female, *M* male, *CINT* high-intensity circuit-type neuromuscular exercise training*, TR* 40 week training group, *TRD* 20 week training – 20 week de-training group, *HIPT* High-Intensity power training, *HICTBW* High-Intensity circuit training using body weight, *CON* control group, *CTL* control group, *HIFT* High-Intensity Functional Training, *FE* free exercise, *VO_2_max* maximal oxygen uptake, *VO_2_peak* peak oxygen uptake, *HR* heart rate, *HRmax* heart rate maximum, *6RM* 6 repetition maximum, *R* right, *L* left, *1RM* 1 repetition maximum, *max* maximum, *BW* body-weight, *CMJ* counter movement jump, *Pmax* maximum power, *PmaxR* relative maximum power, *Pmean* mean power, *PmeanR* relative mean power, *LB* lower body, *UB* upper body, *MB* medicine ball, *PP* peak power, *MP* mean power, ↑ significant improvement, ↔ no significance change, ↓ significant decrease, * significant difference compared to control group

**Electronic Supplementary Table S5b** Detailed results of included studies comparing HIMT vs. structured activity (concurrent training)

| Reference | Training group | Aerobic Fitness | Muscular Fitness | | | Subjective Responses | | | | | | | |  |  |
| --- | --- | --- | --- | --- | --- | --- | --- | --- | --- | --- | --- | --- | --- | --- | --- |
|  |  |  | Muscular Strength | Muscular Endurance | Muscular Power | Exercise enjoyment | Psychological distress | Subjective vitality | Amotivation | External regulation | Introjected regulation | Intrinsic regulation | Identified regulation | HIIT self-efficacy | Autonomous motivation |
| Davis et al. [32] | Integrated CE (M) | ↓ active HR^†^ |  |  |  |  |  |  |  |  |  |  |  |  |  |
|  | Integrated CE (F) | ↑ VO_2_max, ↔ RHR, ↓ active HR^†^ |  |  |  |  |  |  |  |  |  |  |  |  |  |
|  | Serial CE (M) | ↔ active HR |  |  |  |  |  |  |  |  |  |  |  |  |  |
|  | Serial CE (F) | ↑ VO_2_max, ↔ RHR, ↓ active HR |  |  |  |  |  |  |  |  |  |  |  |  |  |
| Davis et al. [33] | Integrated CE |  | ↑ 1RM LB sum^†^, ↑ 1RM UB sum | ↑ Leg press reps^†^, ↔ UB reps |  |  |  |  |  |  |  |  |  |  |  |
|  | Serial CE |  | ↑ 1RM LB sum, ↑ 1RM UB sum | ↔ Leg press reps, ↔ UB reps |  |  |  |  |  |  |  |  |  |  |  |
| Mirzaei et al. [45] | Integrated CE |  | ↑ LB strength, ↑ UB strength | ↑ Trunk endurance | ↑ MB throw, ↑ MB chest throw, ↑ MB supine throw |  |  |  |  |  |  |  |  |  |  |
|  |  |  |  |  |  |  |  |  |  |  |  |  |  |  |  |
|  | Serial CE |  | ↑ LB strength, ↑ UB strength | ↑ Trunk endurance | ↔ MB throw, ↔ MB chest throw, ↔ MB supine throw |  |  |  |  |  |  |  |  |  |  |
|  |  |  |  |  |  |  |  |  |  |  |  |  |  |  |  |
| Heinrich  et al. [24] | CF |  |  |  |  | ↑ |  |  |  |  |  |  |  |  |  |
|  | ART |  |  |  |  | ↑^†^ |  |  |  |  |  |  |  |  |  |
| Carneiro  et al. [35] | HIBWT |  | ↑ R knee extensor, ↑ L knee extensor, ↑ total knee extensor |  |  |  |  |  |  |  |  |  |  |  |  |
|  | COMT |  | ↑ R knee extensor, ↑ L knee extensor†, ↑ total knee extensor^†^ |  |  |  |  |  |  |  |  |  |  |  |  |
| Nunes et al. [42] | BW HIIT |  | ↔ 1RM unilateral leg press , ↔ muscle quality index |  |  |  |  |  |  |  |  |  |  |  |  |
|  | ART |  | ↑ 1RM unilateral leg press^†^ , ↑ muscle quality index^†^ |  |  |  |  |  |  |  |  |  |  |  |  |
| Bahremand  et al. [34] | CF | ↑ VO_2_max | ↑ 1RM squat (estimate), ↑ 1RM bench press (estimate)^†^ |  | Wingate (↑ LBPPO, ↑ LBMPO, ↑ UBPPO, ↔ UBMPO) |  |  |  |  |  |  |  |  |  |  |
|  | CT | ↑ VO_2_max | ↑ 1RM squat (estimate), ↑ 1RM bench press (estimate) |  | Wingate (↑ LBPPO, ↑ LBMPO, ↑ UBPPO, ↔ UBMPO) |  |  |  |  |  |  |  |  |  |  |
| Hovespain  et al. [67] | HIFT | ↑ VO_2_max |  |  | ↔ Vertical jump |  |  |  |  |  |  |  |  |  |  |
|  |  |  |  |  |  |  |  |  |  |  |  |  |  |  |  |
|  | CSCT | ↑ VO_2_max |  |  | ↔ Vertical jump |  |  |  |  |  |  |  |  |  |  |

*RCT* randomised control trial, *HIMT* High-Intensity Multimodal Training, *CE* concurrent exercise, *M* male, *F* female *CF* Crossfit^®^, *ART* aerobic and resistance training, *HIBWT* high-intensity body-weight training, *COMT* combined training, *BW HIIT* body weight high-intensity interval training, *CT* combined training, *HIFT* High-Intensity Functional Training, *CSCT* common strength and conditioning training, *NR* not reported, *VO_2_max* maximal oxygen uptake, *HR* heart rate, *RHR* resting heart rate, *LB* lower body, *UB* upper body, *1RM* 1 repetition maximum, *R* right, *L* left, *MB* medicine ball, *LBPPO* lower body peak power output, *LBMPO* lower body mean power output, *UBPPO* upper body peak power output, *UBMPO* upper body mean power output, ↑ significant improvement, ↔ no significance change, ↓ significant decrease, † significant difference compared structured activity group

**The Effects of High-Intensity Multimodal Training in Apparently Healthy Populations.**

**A Systematic Review.**

Sports Medicine - Open

Tijana Sharp^1^, Clementine Grandou^1^, Aaron J. Coutts^1^, Lee Wallace^1^

^1^Sport and Exercise Discipline Group, University of Technology, Human Performance Research Centre, Moore Park, Sydney, Australia

Corresponding author: Tijana Sharp (tijana.sharp@uts.edu.au)
