# Supplementary material for: The Effects of High-Intensity Multimodal Training in Apparently Healthy Populations: A Systematic Review
Source: Sports Med Open. 2022 Mar 29;8:43. doi: 10.1186/s40798-022-00434-x (PMC8964907; doi:10.1186/s40798-022-00434-x)
Supplement: Supplementary file 6 — Additional file 6. Effect sizes ± 95% confidence intervals. [file 40798_2022_434_MOESM6_ESM.docx]

**Electronic Supplementary Table S6a** Effect sizes ± 95% confidence intervals of pre to post intervention between group changes in studies observing HIMT vs. passive or habitual activity control

|  |  |  | **Effect size (Hedge's g)** | **95% Confidence Interval** | |
| --- | --- | --- | --- | --- | --- |
| **Reference** | **Comparator group** | **Outcome measure** |  | **Lower** | **Upper** |
| **Aerobic Fitness** |  |  |  |  |  |
| Paoli et al. [44] | CHG vs. CG | Submax HR | 1.411 | 0.431 | 2.390 |
| Schmidt et al. [58] | CT-7 vs. CG (M) | VO_2_max | -0.256 | -0.975 | 0.462 |
| Schmidt et al. [58] | CT-7 vs. CG (F) | VO_2_max | 0.773 | 0.110 | 1.436 |
| Schmidt et al. [58] | CT-14 vs. CG (M) | VO_2_max | 0.339 | -0.409 | 1.087 |
| Schmidt et al. [58] | CT-14 vs. CG (F) | VO_2_max | 1.146 | 0.433 | 1.860 |
| Batrakoulis et al. [18] | TR vs. CG | VO_2_max | 3.042 | 2.060 | 4.025 |
| Batrakoulis et al. [18] | TRD vs. CG | VO_2_max | 1.016 | 0.299 | 1.733 |
| Ajjimaporn et al. [43] | HICTBW vs. CG | VO_2_peak (absolute) | 1.745 | 0.805 | 2.685 |
| Ajjimaporn et al. [43] | HICTBW vs. CG | VO_2_peak (relative) | 1.436 | 0.539 | 2.334 |
| Ajjimaporn et al. [43] | HICTBW vs. CG | HR | 0.898 | 0.058 | 1.737 |
| Engel et al. [41] | Functional HIIT vs. CON | HRmax | 0.731 | -0.174 | 1.636 |
| Islam et al. [66] | Tabata vs. CTL | VO_2_peak | 0.000 | -0.635 | 0.635 |
| McWeeny et al. [36] | HIFT vs. FE | VO_2_max | 3.555 | 2.147 | 4.963 |
| **Muscular Fitness** |  |  |  |  |  |
| **Muscular Strength** |  |  |  |  |  |
| Paoli et al. [44] | CHG vs. CG | 6RM bench press | 6.877 | 4.572 | 9.181 |
| Paoli et al. [44] | CHG vs. CG | 6RM leg press | 15.192 | 10.403 | 19.980 |
| Meier et al. [47] | HIIT vs. CG | R grip strength | 0.229 | -0.543 | 1.000 |
| Meier et al. [47] | HIIT vs. CG | L grip strength | 0.196 | -0.574 | 0.967 |
| Schmidt et al. [58] | CT-7 vs. CG (M) | R grip strength | 0.303 | -0.416 | 1.023 |
| Schmidt et al. [58] | CT-7 vs. CG (M) | L grip strength | 0.490 | -0.236 | 1.217 |
| Schmidt et al. [58] | CT-7 vs. CG (F) | R grip strength | -0.050 | -0.689 | 0.590 |
| Schmidt et al. [58] | CT-7 vs. CG (F) | L grip strength | -0.271 | -0.913 | 0.372 |
| Schmidt et al. [58] | CT-14 vs. CG (M) | R grip strength | 0.927 | 0.146 | 1.709 |
| Schmidt et al. [58] | CT-14 vs. CG (M) | L grip strength | 0.012 | -0.731 | 0.754 |
| Schmidt et al. [58] | CT-14 vs. CG (F) | R grip strength | -0.400 | -1.069 | 0.269 |
| Schmidt et al. [58] | CT-14 vs. CG (F) | L grip strength | 0.363 | -0.304 | 1.031 |
| Batrakoulis et al. [18] | TR vs. CG | 1RM leg press | 1.673 | 0.892 | 2.455 |
| Batrakoulis et al. [18] | TRD vs. CG | 1RM leg press | 0.796 | 0.094 | 1.497 |
| Romero-Arenas et al. [46] | HIPT vs. CG | 1RM bench press | 0.286 | -0.595 | 1.167 |
| Romero-Arenas et al. [46] | HIPT vs. CG | 1RM high pull | 0.778 | -0.131 | 1.687 |
| Engel et al. [41] | Functional HIIT vs. CG | Bourban test (ventral) | 0.254 | -0.627 | 1.134 |
| Engel et al. [41] | Functional HIIT vs. CG | Bourban test (R) | 0.636 | -0.262 | 1.535 |
| Engel et al. [41] | Functional HIIT vs. CG | Bourban test (L) | 0.772 | -0.137 | 1.680 |
| McWeeny et al. [36] | HIFT vs. FE | 1RM back squat | 1.151 | 0.204 | 2.097 |
| McWeeny et al. [36] | HIFT vs. FE | 1RM bench press | 0.733 | -0.173 | 1.638 |
| McWeeny et al. [36] | HIFT vs. FE | 1RM leg extension | 4.750 | 3.037 | 6.464 |
| McWeeny et al. [36] | HIFT vs. FE | 1RM pull up | 1.238 | 0.282 | 2.195 |
| McWeeny et al. [36] | HIFT vs. FE | 1RM leg curl | 0.442 | -0.445 | 1.329 |
| Batrakoulis et al. [39] | TR vs. CG | 1RM chest press | 2.095 | 1.260 | 2.931 |
| Batrakoulis et al. [39] | TR vs. CG | 1RM lat pull down | 2.664 | 1.744 | 3.584 |
| Batrakoulis et al. [39] | TR vs. CG | 1RM leg extension | 3.528 | 2.460 | 4.596 |
| Batrakoulis et al. [39] | TR vs. CG | 1RM leg curl | 3.435 | 2.383 | 4.486 |
| Batrakoulis et al. [39] | TRD vs. CG | 1RM chest press | 1.784 | 0.989 | 2.579 |
| Batrakoulis et al. [39] | TRD vs. CG | 1RM lat pull down | 3.031 | 2.051 | 4.012 |
| Batrakoulis et al. [39] | TRD vs. CG | 1RM leg extension | 3.179 | 2.173 | 4.185 |
| Batrakoulis et al. [39] | TRD vs. CG | 1RM leg curl | 2.890 | 1.933 | 3.847 |
| **Muscular Endurance** |  |  |  |  |  |
| Schmidt et al. [58] | CT-7 vs. CG (M) | Push-up | 0.352 | -0.369 | 1.073 |
| Schmidt et al. [58] | CT-7 vs. CG (F) | Push-up | 0.828 | 0.162 | 1.494 |
| Schmidt et al. [58] | CT-14 vs. CG (M) | Push-up | 0.466 | -0.286 | 1.219 |
| Schmidt et al. [58] | CT-14 vs. CG (F) | Push-up | 1.197 | 0.479 | 1.914 |
| Engel et al. [41] | Functional HIIT vs. CG | Leg press | 0.452 | -0.436 | 1.339 |
| Engel et al. [41] | Functional HIIT vs. CG | Chest press | 0.597 | -0.299 | 1.493 |
| Engel et al. [41] | Functional HIIT vs. CG | Pull down | 1.351 | 0.380 | 2.323 |
| Engel et al. [41] | Functional HIIT vs. CG | Back extension | 0.812 | -0.100 | 1.724 |
| Eather et al. [25] | Work-HIIT vs. CG | Push-up | 0.841 | 0.244 | 1.438 |
| Islam et al. [66] | Tabata vs. CTL | Back extension | 0.429 | -0.213 | 1.072 |
| Islam et al. [66] | Tabata vs. CTL | Push-up | 1.272 | 0.579 | 1.965 |
| Islam et al. [66] | Tabata vs. CTL | Sit up | 0.450 | -0.193 | 1.093 |
| Islam et al. [66] | Tabata vs. CTL | R plank | 0.464 | -0.179 | 1.107 |
| Islam et al. [66] | Tabata vs. CTL | L plank | 0.969 | 0.300 | 1.638 |
| McWeeny et al. [36] | HIFT vs. FE | BW squat | -0.198 | -1.077 | 0.680 |
| McWeeny et al. [36] | HIFT vs. FE | Leg extension | 5.732 | 3.751 | 7.713 |
| McWeeny et al. [36] | HIFT vs. FE | Bench press | -0.180 | -1.058 | 0.699 |
| McWeeny et al. [36] | HIFT vs. FE | Leg curl | -0.398 | -1.284 | 0.487 |
| McWeeny et al. [36] | HIFT vs. FE | Bent arm hang | 0.177 | -0.701 | 1.055 |
| Batrakoulis et al. [39] | TR vs. CG | 60s curl up | 6.282 | 4.662 | 7.901 |
| Batrakoulis et al. [39] | TR vs. CG | 60s chair squat | 10.168 | 7.692 | 12.644 |
| Batrakoulis et al. [39] | TR vs. CG | 60s push-up | 4.968 | 3.622 | 6.314 |
| Batrakoulis et al. [39] | TRD vs. CG | 60s curl up | 3.957 | 2.810 | 5.105 |
| Batrakoulis et al. [39] | TRD vs. CG | 60s chair squat | 5.810 | 4.290 | 7.330 |
| Batrakoulis et al. [39] | TRD vs. CG | 60s push-up | 2.443 | 1.557 | 3.329 |
| **Muscular Power** |  |  |  |  |  |
| Romero-Arenas et al. [46] | HIPT vs. CG | PP bench press | 0.611 | -0.285 | 1.508 |
| Romero-Arenas et al. [46] | HIPT vs. CG | PP high pull | 1.463 | 0.476 | 2.450 |
| Romero-Arenas et al. [46] | HIPT vs. CG | CMJ height | 0.681 | -0.220 | 1.583 |
| Romero-Arenas et al. [46] | HIPT vs. CG | CMJ PP | 0.258 | -0.622 | 1.138 |
| Romero-Arenas et al. [46] | HIPT vs. CG | Pmax | 0.777 | -0.132 | 1.686 |
| Romero-Arenas et al. [46] | HIPT vs. CG | PmaxR | 1.410 | 0.431 | 2.390 |
| Romero-Arenas et al. [46] | HIPT vs. CG | Pmean | 0.878 | -0.039 | 1.796 |
| Romero-Arenas et al. [46] | HIPT vs. CG | PmeanR | 1.332 | 0.363 | 2.301 |
| Eather et al. [25] | Work-HIIT vs. CG | Standing jump | 0.618 | 0.033 | 1.204 |
| McWeeny et al. [36] | HIFT vs. FE | CMJ height | -1.037 | -1.971 | -0.104 |
| McWeeny et al. [36] | HIFT vs. FE | MB toss | -2.441 | -3.598 | -1.283 |
| McWeeny et al. [36] | HIFT vs. FE | LBPP | -3.548 | -4.955 | -2.142 |
| McWeeny et al. [36] | HIFT vs. FE | LBMP | -3.101 | -4.402 | -1.800 |
| McWeeny et al. [36] | HIFT vs. FE | UBPP | 1.060 | 0.124 | 1.996 |
| McWeeny et al. [36] | HIFT vs. FE | UBMP | 0.713 | -0.191 | 1.617 |
| **Subjective Responses** |  |  |  |  |  |
| Batrakoulis et al. [40] | TR vs. CG | Psychological distress | 3.434 | 2.383 | 4.485 |
| Batrakoulis et al. [40] | TR vs. CG | Subjective vitality | 1.861 | 1.057 | 2.666 |
| Batrakoulis et al. [40] | TR vs. CG | Amotivation | 0.887 | 0.180 | 1.595 |
| Batrakoulis et al. [40] | TR vs. CG | External regulation | 1.190 | 0.459 | 1.922 |
| Batrakoulis et al. [40] | TR vs. CG | Introjected regulation | 1.690 | 0.907 | 2.474 |
| Batrakoulis et al. [40] | TR vs. CG | Intrinsic regulation | 1.849 | 1.046 | 2.653 |
| Batrakoulis et al. [40] | TR vs. CG | Identified regulation | 4.043 | 2.879 | 5.207 |
| Batrakoulis et al. [40] | TRD vs. CG | Psychological distress | 1.095 | 0.372 | 1.818 |
| Batrakoulis et al. [40] | TRD vs. CG | Subjective vitality | 1.817 | 1.018 | 2.616 |
| Batrakoulis et al. [40] | TRD vs. CG | Amotivation | 0.693 | -0.003 | 1.388 |
| Batrakoulis et al. [40] | TRD vs. CG | External regulation | 0.237 | -0.441 | 0.916 |
| Batrakoulis et al. [40] | TRD vs. CG | Introjected regulation | -0.517 | -1.204 | 0.170 |
| Batrakoulis et al. [40] | TRD vs. CG | Intrinsic regulation | 0.945 | 0.234 | 1.657 |
| Batrakoulis et al. [40] | TRD vs. CG | Identified regulation | 1.334 | 0.589 | 2.079 |
| Eather et al. [25] | Work-HIIT vs. CG | HIIT self-efficacy | 1.656 | 0.993 | 2.319 |
| Eather et al. [25] | Work-HIIT vs. CG | Autonomous motivation | 0.469 | -0.111 | 1.048 |

*HIMT* High-Intensity Multimodal Training*, CHG* circuit high-intensity group, *CG* control group, *CT-7* 7 minute circuit group, *M* male, *F* female, *CT-14* 14 minute circuit group, *TR 40 week training group, TRD* 20 week training – 20 week de-training group*, HICTBW* High-Intensity circuit training with bodyweight, *CON* control group, *CTL* control group, *HIFT* High-Intensity Functional Training, *FE* free exercise, *HIPT* High-Intensity power training, *HR* heart rate, *VO_2_max* maximal oxygen uptake, *VO_2_peak* peak oxygen uptake, *HRmax* heart rate maximum, *6RM* 6 repetition maximum, *R* right, *L* left, *1RM* 1 repetition maximum, *PP* peak power, *CMJ* counter movement jump, *Pmax* maximum power, *PmaxR* relative maximum power, *Pmean* mean power, *PmeanR* relative mean power, *MB* medicine ball, *LBPP* lower body peak power, *LBMP* lower body mean power, *UBPP* upper body peak power, *UBMP* upper body mean power, *HIIT* high-intensity interval training

**Electronic Supplementary Table S6b** Effect sizes ± 95% confidence intervals of pre to post intervention between group changes in studies observing HIMT vs. structured activity (concurrent training)

|  |  |  | **Effect size (Hedge's g)** | **95% Confidence Interval** | |
| --- | --- | --- | --- | --- | --- |
| **Reference** | **Comparator group** | **Outcome measure** |  | **Lower** | **Upper** |
| **Aerobic Fitness** |  |  |  |  |  |
| Davis et al. [32] | Integrated CE vs. Serial CE (M) | VO_2_max | NR | NR | NR |
| Davis et al. [32] | Integrated CE vs. Serial CE (M) | RHR | NR | NR | NR |
| Davis et al. [32] | Integrated CE vs. Serial CE (M) | Submax HR | 0.106 | -0.771 | 0.983 |
| Davis et al. [32] | Integrated CE vs. Serial CE (F) | VO_2_max | 0.535 | -0.250 | 1.320 |
| Davis et al. [32] | Integrated CE vs. Serial CE (F) | RHR | NR | NR | NR |
| Davis et al. [32] | Integrated CE vs. Serial CE (F) | Submax HR | 0.677 | -0.224 | 1.578 |
| Bahremand et al. [34] | CF vs. CT | VO_2_max | 0.980 | 0.221 | 1.739 |
| Hovsepian et al. [67] | HIFT vs. CSCT | VO_2_max | 0.541 | -0.351 | 1.434 |
| **Muscular Fitness** |  |  |  |  |  |
| Davis et al. [33] | Integrated CE vs. Serial CE | LB 1RM | 0.509 | -0.258 | 1.276 |
| Davis et al. [33] | Integrated CE vs. Serial CE | UB 1RM | -0.017 | -0.786 | 0.752 |
| Davis et al. [33] | Integrated CE vs. Serial CE | Leg press repetitions | 0.195 | -0.562 | 0.951 |
| Davis et al. [33] | Integrated CE vs. Serial CE | UB repetitions | -0.304 | -1.077 | 0.469 |
| Mirzaei et al. [45] | Integrated CE vs. Serial CE | LB strength | -0.098 | -0.999 | 0.804 |
| Mirzaei et al. [45] | Integrated CE vs. Serial CE | UB strength | 0.150 | -0.752 | 1.052 |
| Mirzaei et al. [45] | Integrated CE vs. Serial CE | Trunk endurance | -0.557 | -1.475 | 0.360 |
| Mirzaei et al. [45] | Integrated CE vs. Serial CE | MB throw | 0.320 | -0.587 | 1.226 |
| Mirzaei et al. [45] | Integrated CE vs. Serial CE | MB chest throw | 0.081 | -0.819 | 0.982 |
| Mirzaei et al. [45] | Integrated CE vs. Serial CE | MB supine throw | 0.015 | -0.886 | 0.915 |
| Carneiro et al. [35] | HIBWT vs. CT | R knee extensor | -0.699 | -1.523 | 0.124 |
| Carneiro et al. [35] | HIBWT vs. CT | L knee extensor | -1.017 | -1.866 | -0.169 |
| Carneiro et al. [35] | HIBWT vs. CT | Total knee extensor | -0.838 | -1.671 | -0.005 |
| Nunes et al. [42] | HIIT vs. CT | Muscle strength | -0.735 | -1.561 | 0.092 |
| Nunes et al. [42] | HIIT vs. CT | Muscle quality index | -0.060 | -0.861 | 0.740 |
| Bahremand et al. [34] | CF vs. CT | LB strength | -0.564 | -1.296 | 0.167 |
| Bahremand et al. [34] | CF vs. CT | UB strength | 0.873 | 0.123 | 1.624 |
| Bahremand et al. [34] | CF vs. CT | LBPPO | 0.082 | -0.636 | 0.799 |
| Bahremand et al. [34] | CF vs. CT | LBMPO | -0.919 | -1.673 | -0.165 |
| Bahremand et al. [34] | CF vs. CT | UBPPO | -0.537 | -1.267 | 0.193 |
| Bahremand et al. [34] | CF vs. CT | UBMPO | 0.287 | -0.434 | 1.008 |
| Hovsepian et al. [67] | HIFT vs. CSCT | Vertical jump | 0.269 | -0.611 | 1.150 |
| **Subjective Responses** |  |  |  |  |  |
| Heinrich et al. [24] | CF vs. ART | Exercise Enjoyment | 2.709 | 1.430 | 3.989 |

*CE* concurrent exercise, *M* male, *F* female, *CF* CrossFit^®^, *CT* combined training, *HIFT* High-Intensity Functional Training, *CSCT* common strength and conditioning training, *HIBWT* High-Intensity bodyweight training, *ART* aerobic and resistance training, *VO2max* maximal oxygen uptake, *RHR* resting heart rate, *HR* heart rate, *LB* lower body, *UB* upper body, *1RM* 1 repetition maximum, *MB* medicine ball, *R* right, *L* left, *LBPPO* lower body peak power output, *LBMPO* lower body mean power output, *UBPPO* upper body peak power output, *UBMPO* upper body mean power output, *NR* not reported


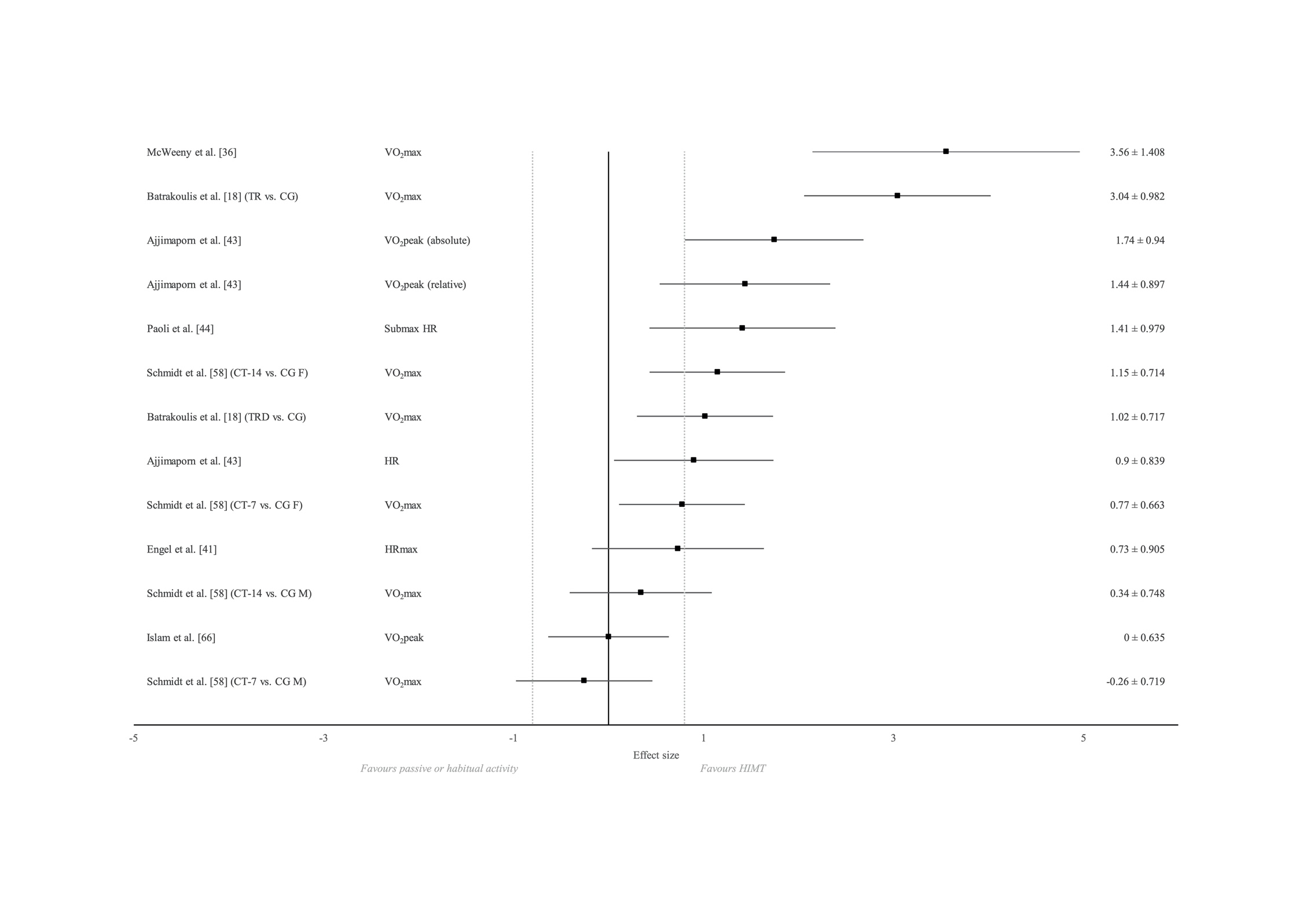


*Favours HIMT*

*Favours non-structured exercise*

**Electronic Supplementary Fig. S1a** Effect sizes ± 95% confidence intervals of pre to post intervention between group changes in aerobic fitness for studies observing HIMT vs. passive or habitual activity control *HIMT* High-Intensity Multimodal Training, *TR* 40 week training group, *TRD* 20 week training: 20 week detraining group, *CT-7* 7 minute circuit training group, *CT-14* 14 minute circuit training group, *CG* control group *F* female, *M* male*, VO_2_max* maximal oxygen uptake, *VO_2_peak* peak oxygen uptake, *HR* heart rate, *HRmax* heart rate maximum


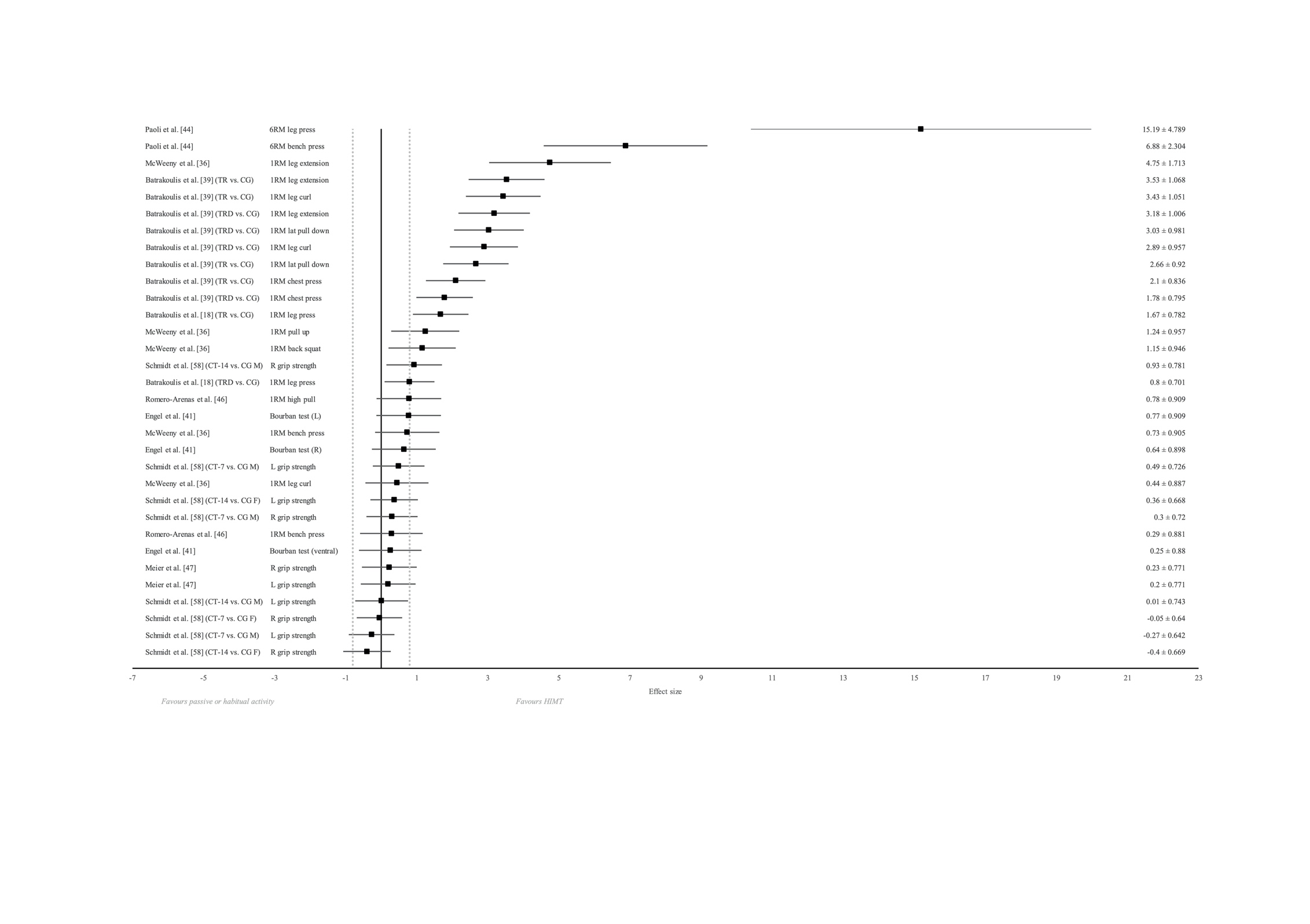


**Electronic Supplementary Fig. S1b** Effect sizes ± 95% confidence intervals of pre to post intervention between group changes in muscular strength for studies observing HIMT vs. passive or habitual activity control *HIMT* High-Intensity Multimodal Training*, TR* 40 week training group, *TRD* 20 week training: 20 week detraining group, *CT-7* 7 minute circuit training group, *CT-14* 14 minute circuit training group, *CG* control group *F* female, *M* male*, 6RM* 6 repetition maximum, *1RM* 1 repetition maximum, *R* right, *L* left

*Favours HIMT*

*Favours non-structured exercise*

*Favours HIMT*

*Favours non-structured exercise*

**
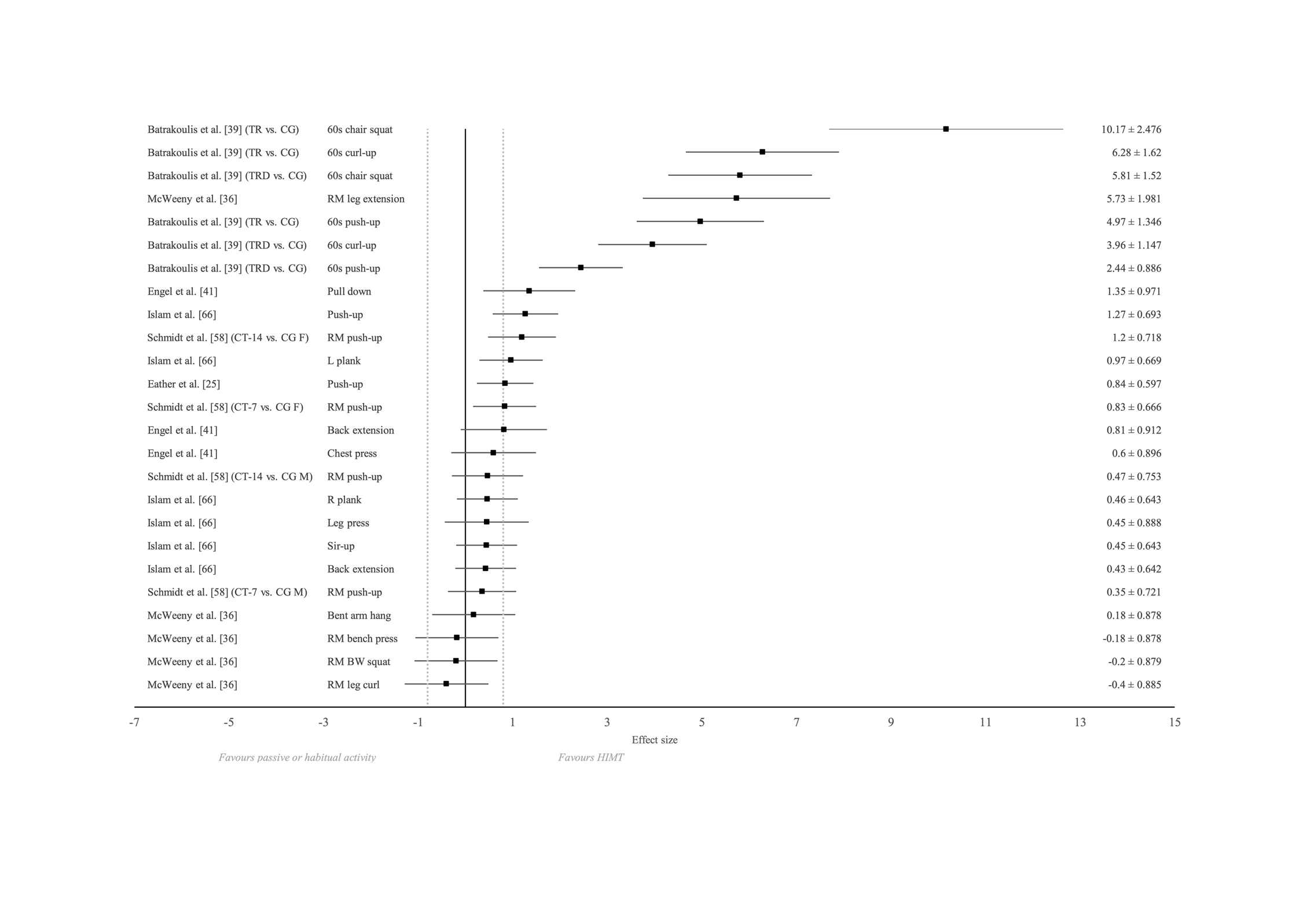
**

**Electronic Supplementary Fig. S1c** Effect sizes ± 95% confidence intervals of pre to post intervention between group changes in muscular endurance for studies observing HIMT vs. passive or habitual activity control *HIMT* High-Intensity Multimodal Training*, TR* 40 week training group, *TRD* 20 week training: 20 week detraining group, *CT-7* 7 minute circuit training group, *CT-14* 14 minute circuit training group, *CG* control group *F* female, *M* male, *RM* repetition maximum, *reps* repetitions, *R* right, *L* left, *BW* bodyweight

*Favours HIMT*

*Favours non-structured exercise*


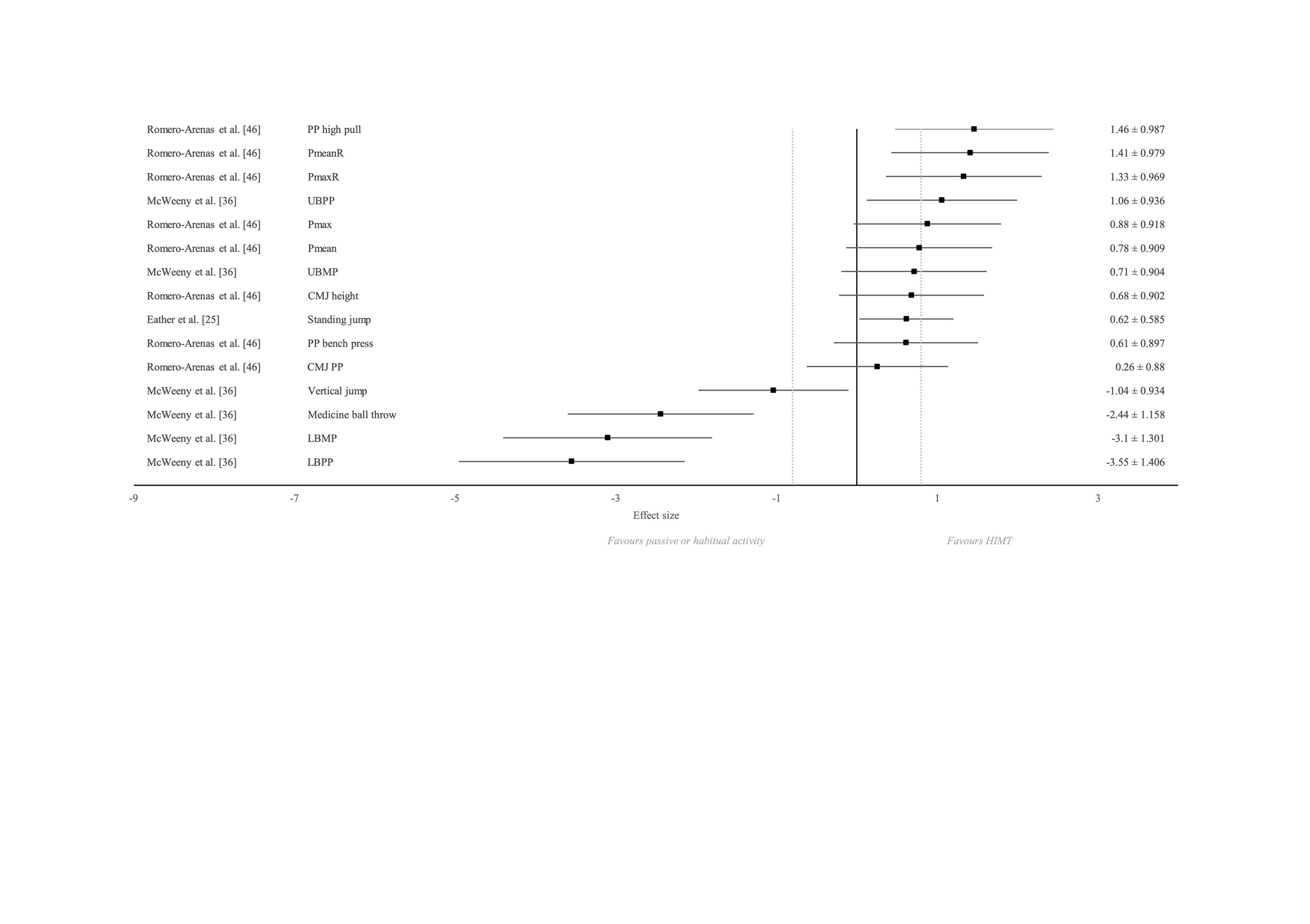


**Electronic Supplementary Fig. S1d** Effect sizes ± 95% confidence intervals of pre to post intervention between group changes in muscular power for studies observing HIMT vs. passive or habitual activity control *HIMT* High-Intensity Multimodal Training*, PP* peak power, *PmeanR* relative mean power, *PmaxR* relative maximum power, *UBPP* upper body peak power, *Pmax* maximum power, *Pmean* mean power, *UBMP* upper body mean power, *CMJ* counter movement jump, *LBMP* lower body mean power, *LBPP* lower body peak power

*Favours HIMT*

*Favours non-structured exercise*


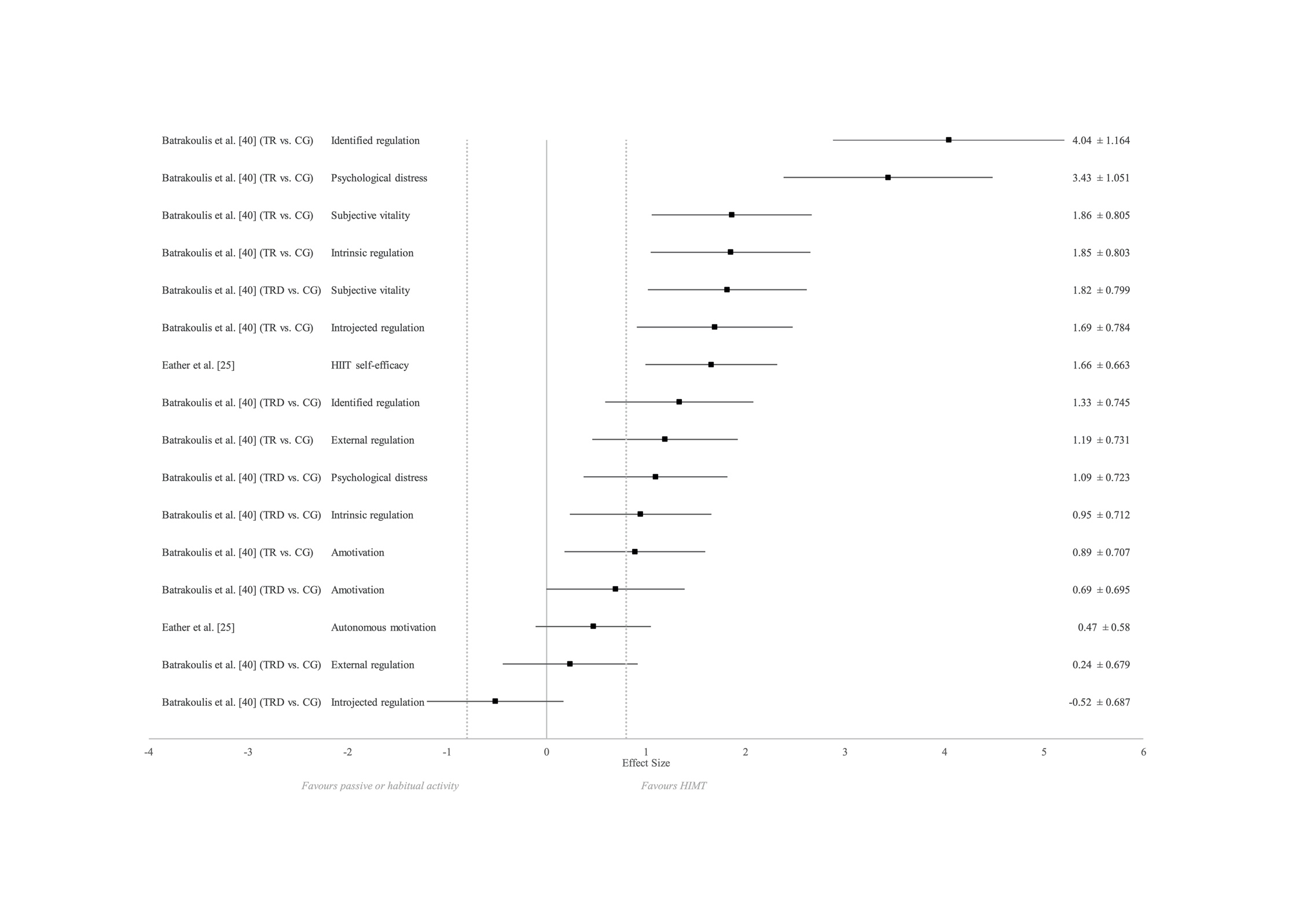


**Electronic Supplementary Fig. S1e** Effect sizes ± 95% confidence intervals of pre to post intervention between group changes in subjective responses for studies observing HIMT vs. passive or habitual activity control *HIMT* High-Intensity Multimodal Training*,* *TR* 40 week training group, *TRD* 20 week training: 20 week detraining group, *CG* control group. *HIIT* high-intensity interval training


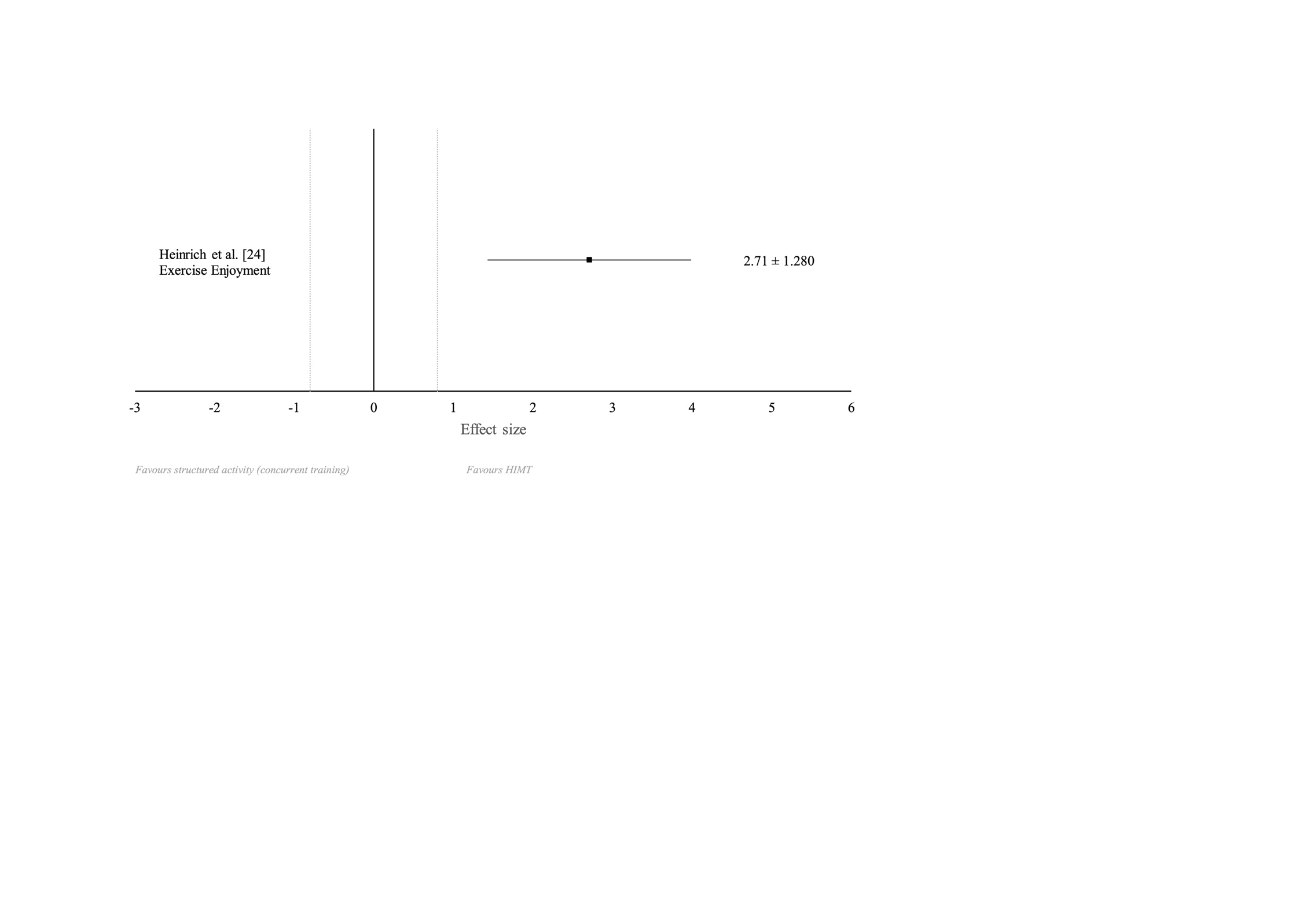


**Electronic Supplementary Fig. S1f** Effect sizes ± 95% confidence intervals of pre to post intervention between group changes in exercise enjoyment for studies observing HIMT vs. structured activity (concurrent training).

**The Effects of High-Intensity Multimodal Training in Apparently Healthy Populations.**

**A Systematic Review.**

Sports Medicine - Open

Tijana Sharp^1^, Clementine Grandou^1^, Aaron J. Coutts^1^, Lee Wallace^1^

^1^Sport and Exercise Discipline Group, University of Technology, Human Performance Research Centre, Moore Park, Sydney, Australia

Corresponding author: Tijana Sharp (tijana.sharp@uts.edu.au)
