# Supplementary material for: The Effects of High-Intensity Multimodal Training in Apparently Healthy Populations: A Systematic Review
Source: Sports Med Open. 2022 Mar 29;8:43. doi: 10.1186/s40798-022-00434-x (PMC8964907; doi:10.1186/s40798-022-00434-x)
Supplement: Supplementary file 7 — Additional file 7. Quality assessment of included studies. [file 40798_2022_434_MOESM7_ESM.docx]

| Reference | Domain A | | | | Domain B | | | | | | | | Domain C | | | | | Domain D | | | | | | Domain E | | | | Overall | |
| --- | --- | --- | --- | --- | --- | --- | --- | --- | --- | --- | --- | --- | --- | --- | --- | --- | --- | --- | --- | --- | --- | --- | --- | --- | --- | --- | --- | --- | --- |
|  | 1.1 | 1.2 | 1.3 | ROB | 2.1 | 2.2 | 2.3 | 2.4 | 2.5 | 2.6 | 2.7^ | ROB | 3.1 | 3.2 | 3.3 | 3.4 | ROB | 4.1 | 4.2 | 4.3 | 4.4 | 4.5 | ROB | 5.1 | 5.2 | 5.3 | ROB | ROB |  |
| *HIMT vs.*  *passive or habitual activity control* | | | | | | | | | | | | | | | | | | | | | | | | | | | |  |  |
| Paoli et al. [44] | NI | NI | PN | - | Y | Y | NI | NI | NI | Y |  | - | Y | NA | NA | NA | + | N | N | NI | PY | PN | - | NI | NI | NI | - | X |  |
| Meier et al. [47] | NI | NI | N | - | Y | Y | NI | NI | NI | PY |  | - | PY | NA | NA | NA | + | N | N | NI | PY | PN | - | NI | NI | NI | - | X |  |
| Schmidt et al. [58] | NI | NI | N | - | Y | Y | NI | NI | NI | N |  | X | N | N | NI | NI | X | N | N | NI | PY | PN | - | NI | NI | NI | - | X |  |
| Batrakoulis et al. [18] | Y | Y | N | + | Y | Y | NI | NI | NI | N |  | X | PN | N | NI | NI | X | N | N | NI | PY | PN | - | NI | N | NI | - | X |  |
| Romero-Arenas et al. [46] | NI | NI | N | - | Y | Y | NI | NI | NI | PY |  | - | PY | NA | NA | NA | + | N | N | PN | NA | NA | + | NI | NI | NI | - | X |  |
| Ajjimaporn et al. [43] | NI | NI | N | - | Y | Y | NI | NI | NI | PY |  | - | Y | NA | NA | NA | + | N | N | NI | PY | PN | - | NI | NI | NI | - | X |  |
| Engel et al. [41] | Y | NI | N | - | Y | Y | NI | NI | NI | PY |  | - | Y | NA | NA | NA | + | N | N | PY | PY | PY | X | NI | NI | NI | - | X |  |
| Batrakoulis et al. [40] | Y | Y | N | + | Y | Y | NI | NI | NI | N |  | X | PN | N | NI | NI | X | N | N | PY | PY | PY | X | NI | N | NI | - | X |  |
| Eather et al. [25]^a^ | Y | NI | N | - | Y | Y | PN | NA | NA | Y | NA | + | PN | N | NI | NI | X | N | N | PY | PY | PY | X | NI | N | NI | - | X |  |
| Islam et al. [66] | NI | NI | N | - | Y | Y | NI | NI | NI | N |  | X | PN | N | NI | NI | X | PN | N | NI | PY | PN | - | NI | NI | NI | - | X |  |
| McWeeny et al. [36] | NI | NI | N | - | Y | Y | NI | NI | NI | PY |  | - | Y | NA | NA | NA | + | N | N | NI | PY | PN | - | NI | NI | NI | - | X |  |
| Batrakoulis et al. [39] | Y | Y | N | + | Y | Y | NI | NI | NI | N |  | X | PN | N | NI | NI | X | N | N | NI | PY | PN | - | NI | N | NI | - | X |  |
| *HIMT vs. structured activity (combined training)* | | | | | | | | | | | | | | | | | | | | | | | | | | | | |  |
| Davis et al. [32] | PN | NI | PY | X | PN | Y | NI | NI | NI | N |  | X | N | N | NI | NI | X | N | N | PN | NA | NA | + | NI | NI | NI | - | X |  |
| Davis et al. [33] | PN | NI | PY | X | PN | Y | NI | NI | NI | N |  | X | PN | PN | NI | NI | X | N | N | PN | NA | NA | + | NI | NI | NI | - | X |  |
| Mirzaei et al. [45] | NI | NI | N | - | Y | Y | NI | NI | NI | PY |  | - | PY | NA | NA | NA | + | N | N | NI | PY | PN | - | NI | NI | NI | - | X |  |
| Heinrich et al. [24] | NI | NI | N | - | Y | Y | NI | NI | NI | N |  | X | N | N | PY | PY | X | PN | N | PY | PY | PY | X | N | NI | NI | - | X |  |
| Carneiro et al. [35] | NI | NI | N | - | Y | Y | NI | NI | NI | N |  | X | N | N | NI | NI | X | N | N | NI | PY | PN | - | NI | NI | NI | - | X |  |
| Nunes et al. [42] | PY | NI | N | - | Y | Y | NI | NI | NI | PY |  | - | Y | NA | NA | NA | + | PN | N | NI | PY | PN | - | NI | PN | NI | - | X |  |
| Bahremand et al. [34] | NI | NI | N | - | Y | Y | NI | NI | NI | N |  | X | NI | N | NI | NI | X | N | N | NI | PY | PN | - | NI | NI | NI | - | X |  |
| Hovsepian et al. [67] | NI | NI | N | - | Y | Y | NI | NI | NI | N |  | X | NI | N | NI | NI | X | N | N | NI | PY | PN | - | NI | NI | NI | - | X |  |

**Electronic Supplementary Table S7** Quality assessment of included studies

*HIMT,* High-Intensity Multimodal Training; *Domain*: *A* risk of bias arising from the randomisation process, *B* risk of bias due to deviations from the intended interventions: *1) effects of assignment to intervention for intention to treat analysis; 2) effects of adhering to intervention for the ‘per-protocol effect, C* risk of bias due to missing outcome data, *D* risk of bias in measurement of the outcome, *E* risk of bias in the selection of the reported result, *ROB* risk of bias, *NI* no information, *Y* yes, *N* no, *NA* not applicable, + low risk of bias, - some concerns, X high risk of bias, ^a^ intention to treat analysis

**Electronic Supplementary Fig. S2a** Summary quality assessment of included studies (effects of assignment to intervention)

**Electronic Supplementary Fig. S2b** Summary quality assessment of included studies (effects of adhering to intervention)

**The Effects of High-Intensity Multimodal Training in Apparently Healthy Populations.**

**A Systematic Review.**

Sports Medicine - Open

Tijana Sharp^1^, Clementine Grandou^1^, Aaron J. Coutts^1^, Lee Wallace^1^

^1^Sport and Exercise Discipline Group, University of Technology, Human Performance Research Centre,

Moore Park, Sydney, Australia

Corresponding author: Tijana Sharp (tijana.sharp@uts.edu.au)
